# Intracellular osteopontin protects from autoimmunity-driven lymphoma development inhibiting TLR9-MYD88-STAT3 signaling

**DOI:** 10.1186/s12943-022-01687-6

**Published:** 2022-12-12

**Authors:** Celeste Rizzello, Valeria Cancila, Sabina Sangaletti, Laura Botti, Chiara Ratti, Matteo Milani, Matteo Dugo, Francesco Bertoni, Claudio Tripodo, Claudia Chiodoni, Mario P. Colombo

**Affiliations:** 1 Molecular Immunology Unit, Department of Research, Fondazione IRCCS Istituto Nazionale Tumori, Via Venezian 1, 20133 Milan, Italy; 2 Tumor Immunology Unit, Department of Health Science, University of Palermo School of Medicine, Palermo, Italy; 3 Platform of Integrated Biology, Department of Applied Research and Technology Development, Fondazione IRCCS Istituto Nazionale Tumori, Via Venezian 1, 20133 Milan, Italy; 4 Department of Medical Oncology, IRCCS Ospedale San Raffaele, Via Olgettina 60, 20132 Milan, Italy; 5 Institute of Oncology Research, Faculty of Biomedical Sciences, USI, Via F. Chiesa 5, 6500 Bellinzona, Switzerland; 6 Oncology Institute of Southern Switzerland, Ente Ospedialiero Cantonale, Via A. Gallino 12, 6500 Bellinzona, Switzerland; 7 FIRC Institute of Molecular Oncology (IFOM), Milan, Italy

**Keywords:** Osteopontin, Diffuse large B cell lymphoma, Autoimmunity

## Abstract

**Background:**

Autoimmune disorders, including Systemic Lupus Erythematosus (SLE), are associated with increased incidence of hematological malignancies. The matricellular protein osteopontin (OPN) has been linked to SLE pathogenesis, as SLE patients show increased serum levels of OPN and often polymorphisms in its gene. Although widely studied for its pro-tumorigenic role in different solid tumours, the role of OPN in autoimmunity-driven lymphomagenesis has not been investigated yet.

**Methods:**

To test the role of OPN in the SLE-associated lymphomagenesis, the SLE-like prone Fas^lpr/lpr^ mutation was transferred onto an OPN-deficient background. Spleen from Fas^lpr/lpr^ and OPN-/-Fas^lpr/lpr^ mice, as well as purified B cells, were analysed by histopathology, flow cytometry, Western Blot, immunohistochemistry, immunofluorescence and gene expression profile to define lymphoma characteristics and investigate the molecular mechanisms behind the observed phenotype. OPN cellular localization in primary splenic B cells and mouse and human DLBCL cell lines was assessed by confocal microscopy. Finally, gain of function experiments, by stable over-expression of the secreted (sOPN) and intracellular OPN (iOPN) in OPN-/-Fas^lpr/lpr^ -derived DLBCL cell lines, were performed for further validation experiments.

**Results:**

Despite reduced autoimmunity signs, OPN-/-Fas^lpr/lpr^ mice developed splenic lymphomas with higher incidence than Fas^lpr/lpr^ counterparts. In situ and ex vivo analysis featured such tumours as activated type of diffuse large B cell lymphoma (ABC-DLBCL), expressing BCL2 and c-MYC, but not BCL6, with activated STAT3 signaling. OPN-/-Fas^lpr/lpr^ B lymphocytes showed an enhanced TLR9-MYD88 signaling pathway, either at baseline or after stimulation with CpG oligonucleotides, which mimic dsDNA circulating in autoimmune conditions. B cells from Fas^lpr/lpr^ mice were found to express the intracellular form of OPN. Accordingly, gene transfer-mediated re-expression of iOPN, but not of its secreted isoform, into ABC-DLBCL cell lines established from OPN-/-Fas^lpr/lpr^ mice, prevented CpG-mediated activation of STAT3, suggesting that the intracellular form of OPN may represent a brake to TLR9 signaling pathway activation.

**Conclusion:**

These data indicate that, in the setting of SLE-like syndrome in which double strand-DNA chronically circulates and activates TLRs, B cell intracellular OPN exerts a protective role in autoimmunity-driven DLBCL development, mainly acting as a brake in the TLR9-MYD88-STAT3 signaling pathway.

**Supplementary Information:**

The online version contains supplementary material available at 10.1186/s12943-022-01687-6.

## Introduction

Autoimmune disorders affect 5–8% of the population worldwide, with approximately 75% of patients being women [1]. In the pathogenesis of Systemic lupus erythematosus (SLE), the chronic IgG antibody production directed at ubiquitous self-antigens released from injured tissues leads to a continuous formation of immune-complexes that promote tissue damage [2].

Over the years, several studies have pointed out that certain autoimmune and chronic inflammatory conditions, including rheumatoid arthritis, Sjögren's syndrome, SLE, psoriasis and celiac disease are associated with increased occurrence of hematological malignancies [3–5]. In particular, SLE patients have a higher risk than healthy people of developing lymphoma, mostly diffuse large B cell lymphoma (DLBCL) [4]. Such a link may be due to the chronic inflammatory state induced by the persistent circulation of self-antigens that directly sustains B cell proliferation and response, thus increasing the possibility of transforming events.

DLBCL, the most common type of NHL worldwide, is a heterogeneous disease in terms of molecular pattern and clinical manifestations and response to therapy [6]. According to the most common classification referred to the cell of origin, DLBCLs are subdivided into germinal center B cell like type (GCB) and activated B-cell like (ABC) subtypes, while about 10–15% of cases are unclassifiable [7]. Additional information is offered by c-MYC and BCL2 status, which further classify “double hit” (DHLs) and “double expressor” (DELs) lymphomas, according to whether these molecules are genetically rearranged or overexpressed without translocations, respectively [8].

We have previously demonstrated that the deficiency of another matricellular protein, SPARC, in Fas^lpr/lpr^ mice induces the disarrangement of the secondary lymphoid organ (SLO) stromal network and exacerbated autoimmunity. This condition results in the development of a CD5 + B cell lymphoid malignancy mimicking human CLL [9]. These results showing the importance of SLO stromal microenvironment in the regulation of autoimmunity-related lymphomagenesis prompted us to investigate the effects of OPN, a different matricellular protein, already associated to autoimmunity. Indeed, this molecule acts both as matricellular protein, interplaying with fibronectin and collagen, thereby regulating matrix connections and cell adhesion, and as a cytokine, binding different integrins and CD44 and promoting processes like cell survival, adhesion and migration [10]. Indeed, sOPN has been demonstrated to be a pro-tumorigenic protein, both in vitro and in vivo mouse models [11–13] and high level of serum/plasma OPN can be detected in cancer patients [14, 15]. In the mouse, a secreted (sOPN) and an intracellular osteopontin (iOPN) isoforms have been characterized. The intracellular one derives from the translation starting at a non-AUG codon located downstream the canonical one, therefore deleting the sequence forwarding to the secretory vesicles [16] intracellular OPN acts as an adaptor molecule in the Toll-like receptor (TLR)-MYD88 pathway, but its final effect seems opposite depending on the cell type. While in plasmacytoid dendritic cells iOPN has been shown to promote TLR9-mediated IFN-α production [17], in macrophages it was found to negatively control the inflammatory response triggered by cellular debris [18] or LPS [19].

In humans, despite not clearly characterized as in the mouse, the presence of a nuclear OPN was observed in epithelial 239 T cells [20] and in the Rck8 DLBCL lymphoma cell line [21], being positively associated with cell cycle progression and NF-κB activity, respectively.

OPN is promptly upregulated in the inflamed tissues and serum of SLE patients as well as in the plasma of Fas^lpr/lpr^ mice, but little is known about OPN contribution to lymphoid malignancies, except from being up-regulated in primary central nervous system lymphomas (PCNSL) [21] and in bone marrow blasts of acute myeloid leukemia patients [22], and promoting the dormancy of acute lymphoblastic leukemia clones in the bone marrow osteoblastic niche [23].

As the autoimmunity-prone Fas^lpr/lpr^ mouse strain spontaneously develops lymphomas late in life [24], we took advantage of this model to study the role of OPN in autoimmunity-driven B cell transformation. We generated OPN-/-Fas^lpr/lpr^ mice and evaluated the dynamics of B cell malignant transformation in these animals in comparison with OPN-competent Fas^lpr/lpr^ mice.

## Methods

### Animals

Eight-week old BALB/c and *nu/nu* mice were purchased from Charles River Laboratories. C57BL/6 *spp1*^−/−^ mice (B6.129S6(Cg)-Spp1^tm1Blh^/J were purchased from the Jackson laboratory and backcrossed on BALB/c background for 10 generations, obtaining BALB/c;Spp1^tm1Blh^ animals [25]. MRL/MpJ-Fas^lpr^/J mice were originally purchased from the Jackson Laboratory and crossed with BALB/c mice for 10 generations to obtain BALB/c;Tnfrsf6lpr strain (referred as Fas^lpr/lpr^) [9]. Fas^lpr/lpr^ mice were backcrossed with BALB/c;Spp1^tm1Blh^, obtaining OPN-/-Fas^lpr/lpr^ animals. Mice were maintained as heterozygous Fas^lpr/+^ for breeding to avoid development of autoimminity signs. Autoimmune mice (Fas^lpr/lpr^ and OPN-/-Fas^lpr/lpr^) were sacrificed for autoimmunity and lymphoma analyses between 4 and 6 months. All animal experiments were approved by the Institutional Ethics Committee for Animal Use and by the Italian Ministry of Health (authorization numbers 1026/2016-PR and 419/2021-PR) and were performed following to the 3Rs’ recommendations (Reduction, Refinement and Replacement).

### Cell lines

The lymphoma cell lines OPL239 and OPL241 were established in our laboratory by in vitro seeding of cell suspension from the lymphomatous spleens of two 6 month-old OPN-/-Fas^lpr/lpr^ mice. Cell lines were cultured in RPMI-1640 (Lonza, #12-115F), supplemented with 10% FBS (Euroclone, #ECS0180L), non-essential amminoacid (NEAA) mixture (Lonza, #13-114E), Hepes (Lonza, #17-737E), Na pyruvate (Lonza, #BE13-115E) and 50 μM β-mercaptoethanol (Sigma, #M7522). The human DLBCL cell lines SU-DHL-4, SU-DHL-10, TMD8 and U2932 were kindly provided by Stefano Casola, from IFOM Institute (Milan) and maintained in culture in RPMI-1640 medium, as above, supplemented with 20% FBS. To confirm tumorigenicity, OPL239 and OPL241 cell lines were intravenously injected into *nu/nu* female mice at the dose of 5 × 10^5^ cells.

### Evaluation of autoimmunity in Fas^lpr/lpr^ and OPN-/-Fas^lpr/lpr^ mice

The percentage of autoimmune CD3 + B220 + T cells was calculated by flow cytometry analysis on cell suspension from spleen and blood samples. Histopathological evaluation of spleen architecture was performed by a pathologist.

### ELISA on mouse sera

The serum was isolated from the blood through a thermal shock (incubation at 37 °C for 30 min and at 4 °C for 1 h) followed by centrifugation at 13,000 rpm for 1 min. Supernatants (sera) were stored at -20 °C. The DuoSet kit for mouse Osteopontin (R&D System, #DY441) was used to test the amount of secreted OPN, following manufacturer’s instructions.

### Evaluation of lymphoma by histopathology

Autoimmune mice were sacrificed at 4–6 months of age and hematoxylin and eosin (H&E) staining was performed on formalin-fixed paraffin-embedded **(**FFPE) spleen sections to evaluate the presence of lymphomatous cells**.** Histopathological analyses were performed according to the criteria for lymphoid neoplasm classification by the pathologist [26].

### Flow cytometry analysis

Spleens were mechanically disaggregated using the bottom of a syringe, treated with ACK (Ammonium-Chloride-Potassium) solution to lyse red blood cells and cell suspensions stained with a mixture of antibodies to characterize splenic immune cells and DLBCL cell lines. Samples were acquired on a LSRFortessa flow cytometer (BD) and analysed using FlowJo v10 software. See Supplementary Material and Methods for the list of antibodies for flow cytometry.

### Immunohistochemistry and immunofluorescence on murine and human tissues

Human tissue samples were collected according to the Helsinki Declaration and the study was approved by the University of Palermo Ethical Review Board (Approval number 05/2018 and 06/2021). Four-micrometers-thick sections were deparaffinized, rehydrated and unmasked using Novocastra Epitope Retrieval Solutions, either pH6 (Leica Biosystem, #RE7113-CE), or pH9 (Leica Biosystem, #RE7119-CE) in thermostatic bath at 98 °C for 30 min. After washing, slides underwent neutralization of the endogenous peroxidase with Novocastra Peroxidase Block (Leica Biosystems, #RE7101), and Fc bloking with Novocastra protein block (Leica Biosystems, #RE7102). Then, samples were incubated with primary antibodies (listed in Supplementary Material and Methods). After incubation with HRP-conjugated IgG (H&L) specific secondary antibodies (Life Technologies) or polymer detection kit (Novocastra, Leica Biosystems), signals were revealed with Novocastra DAB Chromogen (Leica Biosystems, #RE7105). Anti-mouse and anti-rabbit Alexa Fluor 488- and 568-conjugated secondary antibodies were used for fluorescence detection. Details on image acquisition and analysis are provided in Supplementary Material and Methods.

### Gene Expression Profile on mouse CD19 + cells

We collected spleens from 5-month-old Fas^lpr/lpr^ mice (without signs of lymphoma) and OPN-/-Fas^lpr/lpr^ mice (that showed initial lymphomatous foci), and, as controls, age-matched BALB/c and OPN-/- mice. CD19 + cells were purified through immunomagnetic beads anti-CD19 (Miltenyi, #130–121-301) by two rounds of purification on column. Purity of B cells was assessed by flow cytometry using antibodies to CD19 and CD3, to exclude relevant contamination from either T cells, or in case of autoimmune mice, CD3 + B220 + double positive cells. A purity of at least 97.5% was obtained for eache sample.

RNA extraction was performed using Quick-RNA Microprep (Zymoresearch, #R1051) and its purity and yield were assessed using NanoDrop 2000c spectrophotometer (Thermofisher scientific). RNA samples were processed for microarray hybridization by the Platform of Integrated Biology Core Facility at Fondazione IRCCS Istituto Nazionale dei Tumori, Milan. For details see Supplementary Material and Methods. Functional annotation was done using Gene Set Enrichment Analysis (GSEA) on fold-change pre-ranked lists with gene sets from the MSigDB collection [27] and the SignatureDB [28], applying as thresholds P and FDR values < 0.05.

The dataset described in this work is available in the GEO repository, with accession number GSE193790 (https://www.ncbi.nlm.nih.gov/geo/query/acc.cgi?acc=GSE193790).

### RNA SCOPE

RNA scope on fresh murine sections was performed using RNAscope 2.5 HD Detection Reagent-BROWN (Advanced Cell Diagnostic) in accordance with the manufacturer’s protocol. Mouse *Prdm1* (#441,871) was used as probe. Novocastra DAB Chromogen (Leica Biosystems, #RE7105) in its Novocastra DAB Substrate Buffer (Leica Biosystems, #RE7106) was used to reveal signals.

### RT-qPCR

RNA extraction was performed using Quick-RNA Microprep (Zymoresearch, #R1051). RNA was reverse transcribed using High Capacity cDNA Reverse Transcription Kit (Thermofisher, #4,368,814); the 20 µl reaction for RT-qPCR was prepeared using TaqMan Fast Universal PCR Master Mix no Amperase UNG (Thermofisher, #4,352,042) and run on a QuantStudio 3 instrument (Thermofisher). Probes used are listed in Supplementary Material and Methods. Gene expression levels were normalized through the comparison with mouse *Gapdh* expression. The expression values are referred as 2^−ΔCT^.

### Evaluation of MYD88 signaling pathway in mouse CD19 + cells and DLBCL cell lines

CD19 + cells were isolated from mouse spleens using Microbeads anti-CD19 + (Miltenyi, #130–121-301) and the purity of cells was assessed by flow cytometry using an antibody against CD19 (CD19 FITC (BD, clone CL1D3, #557,398). For western blot analysis, 2 × 10^6^ cells were seeded in 6-well plates and stimulated for 1 h. For flow cytometry analysis and RT-qPCR 1 × 10^6^ cells were seeded in 12-well plates and stimulated for 3 days. The following TLR agonists were used: the TLR4 agonist LPS (Lipopolysaccharides from Escherichia coli O111:B4, Sigma, #L4130) (1 µg/ml), the TLR9 agonist CpG 1826 (TriLink Biotechnologies, #I66-G01A) (5 µg/ml), the TLR3 agonist Poly I:C (Polycytidylic-inosinic acid potassium salt, Sigma, #P1038) (5 µg/ml). After stimulation cells were collected and washed in PBS. Dry pellets were stored at -80 °C for western blot analysis, while fresh samples were stained with the different antibodies for flow cytometry analysis of activation (FITC-CD19, BD, clone CL1D3, #557,398 and PE-CD86, eBioscience, clone GL1, #12–0862-82).

To evaluate TLR9-MYD88 cascade in OPL239/OPL241 DLBCL cell lines, 1,5 × 10^6^ cells were seeded in 6-well plates and stimulated with 5 μg/ml CpG for 30 min for western blot and 3 h for RT-PCR. Cells were washed in PBS and dry pellet stored at -80 °C for analysis.

### Western blot analysis

Cells were lysed in ice for 20 min using RIPA buffer, added with phosphatase (Phosphostop, Roche, #04,906,837,001) and protease (Complete, #11,836,153,001) inhibitor cocktails, and 1 mM PMSF. Lysates were then centrifuged at 13,000 rpm for 20 min and the supernatants stored at -80 °C. Proteins were quantified through Pierce BCA Protein Assay Kit (Thermofisher, #23,225) and optical density evaluated by Spark Multimode microplate reader. An equal amount of proteins (20 µg) was run on NuPAGE 4–12% pre-casted minigels (Invitrogen, #NP0321BOX) and proteins were transferred to 0.45 μm nitrocellulose membrane (Amersham, #10,600,002). Nonspecific binding sites were blocked in PBS containing 0.1% Tween 20 (Sigma, #P2287-500ML) and 5% BSA (Sigma, #A7906-100G).

Membranes were incubated overnight at 4 °C with the different antibodies listed in Supplementary Material and Methods. After incubation, membranes were washed three times, for 10 min each, in PBS containing 0.1% Tween 20, and then incubated for 30 min at room temperature with the following secondary antibodies: Rabbit anti-goat (Invitrogen, #811,620) and Donkey anti-rabbit (Invitrogen, #A16035). After washing, blots were developed with Clarity Western ECL Substrate (Biorad, #1,705,061) and images were acquired using Chemidoc XRS system (Biorad). Quantification was done with ImageJ 64 software.

### Vector construction, lentiviral particle production and cell line infection

Lentiviral vectors containing the sequences encoding either for the full-length isoform, which encodes the secreted OPN (sOPN) (vector Spp1-IRES Green), or the intracellular isoform (vector iOPN-IRES Green) of mouse OPN, were obtained by cloning the Spp1 full-length sequence (from pUC57-Spp1 plasmid, from DBA Italia) or the iOPN sequence (kind gift from ML. Shinohara, Durham, North Carolina) into the lentiviral backbone pLVX-IRES-ZsGreen1. The empty pLVX-IRES-ZsGreen1 was used as control. For details on the production of lentiviral particles see Supplementary Material and Methods.

### Injection of OPL239 cell variants in BALB/c mice

Female BALB/c mice were sublethally irradiated using “RS 2000 RAD Source Technologies” instrument, with a dose of 5 Gy. Four hours after irradiation, 1 × 10^5^ cells cells were resuspended in 200 µl of 0.9% NaCl and intravenously injected into the tail vein. About 10 days after the injection, at first signs of distress, mice were sacrificed and spleen and liver collected for histopathology and flow cytometry analysis.

### Statistical analysis

Value differences between two groups were assessed by two tails Student *t* test. Differences among more than two groups was evaluated through ordinary one-way ANOVA. When values were associated with two categorical variables, two-way ANOVA was used to assess differences among two or more groups. Differences were considered statistically significant at *p* value (*p*) < 0.05.

## Results

### Autoimmunity severity of Fas^lpr/lpr^ mice is reduced in absence of OPN

We tested whether circulating levels of OPN increase along disease progression in autoimmune-prone Fas^lpr/lpr^ mice, as occurring in SLE patients. ELISA performed at different time points showed increased release of OPN from 2 months of age, at initial signs of autoimmunity, to 5 months, timing of overt autoimmunity (Fig. S1A). As the Fas^lpr/lpr^ mutation allows the expansion, in SLOs, of CD3 + B220 + auto-reactive T cells deriving from precursors unable of apoptosis in the thymus [29], we evaluated the percentage of this population as a parameter of the autoimmune severity and found a higher expansion of CD3 + B220 + cells in Fas^lpr/lpr^ than OPN-/-Fas^lpr/lpr^ spleens (Fig. S1B), associated with exacerbated splenomegaly (Fig. S1C). In agreement, histopathological evaluation of mouse spleens revealed a marked expansion of the white pulp due to the massive infiltration of pleomorphic lymphoid cells with irregular nuclei and variable size: from small to medium cells (autoreactive lymphocytes) in Fas^lpr/lpr^ mice. These nuclei irregularities were less evident in OPN-deficient animals, which instead showed initial lymphomatous foci (Fig. S1D). The reduced expansion of autoimmune CD3 + B220 + cells in the spleen was confirmed also in the peripheral blood of OPN-/-Fas^lpr/lpr^ mice (Fig. S1E).

These data showing reduced autoimmunity in the absence of OPN are in line with its role in favoring the pathogenesis of inflammatory/autoimmune disorders.

### OPN-/-Fas^lpr/lpr^ mice show a higher incidence of lymphomas than Fas^lpr/lpr^ mice

**Fig. 1:**
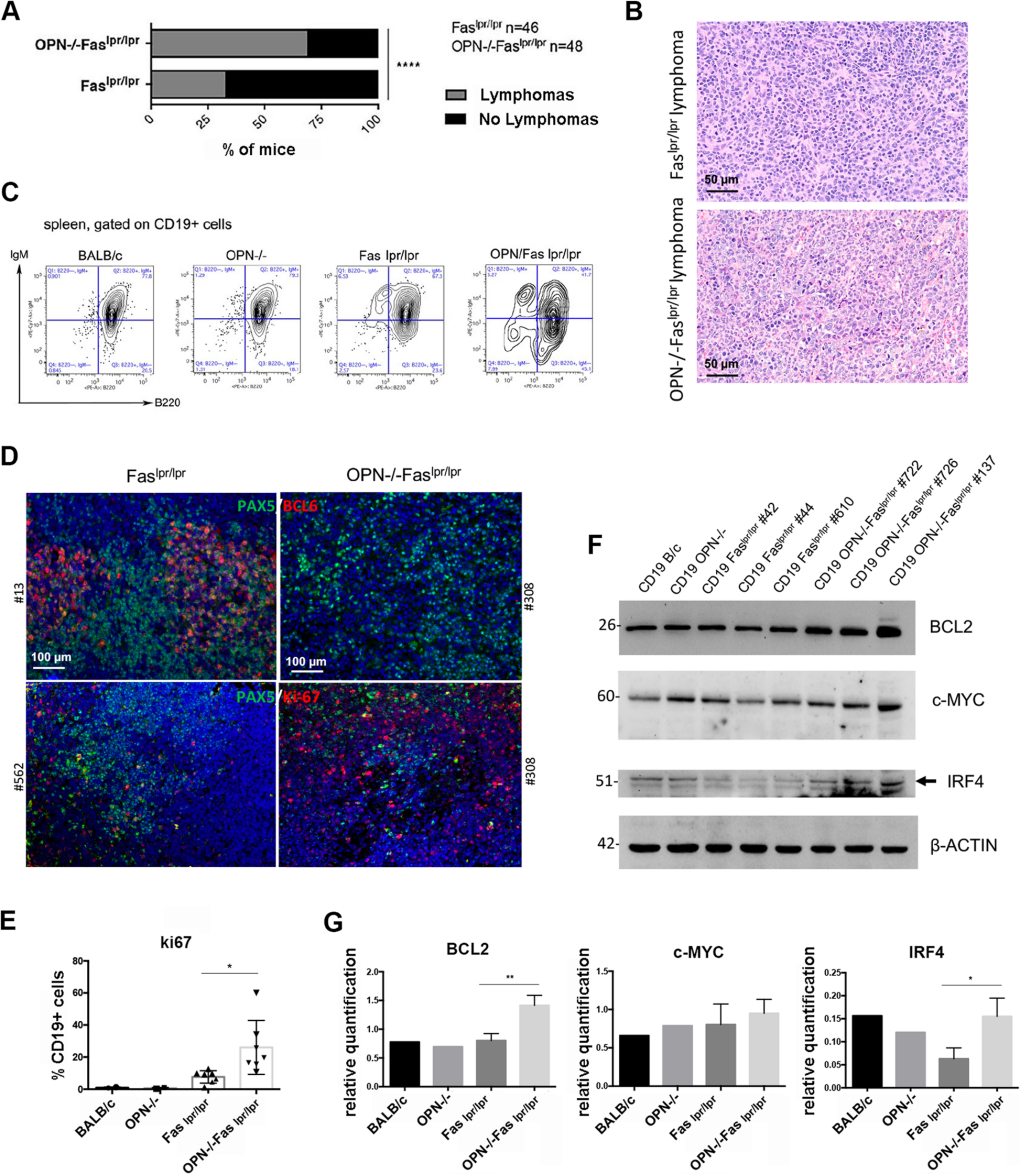
Characterization of lymphomas occurring in Fas^lpr/lpr^ and OPN-/-Fas^lpr/lpr^ mice. **A**. Graph showing the incidence of splenic lymphoma in Fas^lpr/lpr^ (*n = *46) and OPN-/-Fas^lpr/lpr^ (*n = *48) mice at about 5–6 months of age (69% and 33% and respectively) (****, *p <* 0.0001; Chi-square two-tailed test). **B**. Representative H&E staining of Fas^lpr/lpr^ and OPN-/-Fas^lpr/lpr^ tumours. Scale bar: 50 μm. **C**. An example of flow cytometry analysis showing the expression of surface IgM and B220 on CD19 + B cells from naïve and autoimmune mice. **D**. IF for BCL6 (upper panel) and Ki67 (lower panel) on splenic lymphomas from Fas^lpr/lpr^ and OPN-/-Fas^lpr/lpr^ mice. PAX5 is used as pan B cell marker. Scale bar: 100 μm. **E**. Flow cytometry quantification of CD19 + Ki67 + cells in the spleen from Fas^lpr/lpr^ (*n = *7) and OPN-/-Fas^lpr/lpr^ animals (*n = *7) (Student t test; *, p: 0.0153). The graph shows a pool of two independent experiments. **F**. Western blot illustrating the expression of BCL2, c-MYC and IRF4, in splenic CD19 + B cells from Fas^lpr/lpr^ (*n = *3) and OPN-/-Fas^lpr/lpr^ (*n = *3) mice. CD19 + cells from a BALB/c and a OPN-/- mouse were used as controls. **G**. Quantification of western blot, relative to β-ACTIN as housekeeping gene, of BCL2 (Student t test; **, p: 0.0076), c-MYC (Student t test; ns, p: 0.4781) and IRF4 (Student t test; *, *p*: 0.0260). Statistics was applied to values related to Fas^lpr/lpr^ and OPN-/-Fas^lpr/lpr^ B cells

To evaluate the impact of OPN in the autoimmunity-driven lymphomagenesis that characterises aged Fas^lpr/lpr^ mice [24], we compared the incidence of splenic lymphomas in Fas^lpr/lpr^ and OPN-/-Fas^lpr/lpr^ of 5–6 months of age. Differently from the degree of autoimmunity, the incidence of lymphomas was higher in OPN-/-Fas^lpr/lpr^ mice than in Fas^lpr/lpr^ animals [69% and 33% of cases, respectively (Fig. 1A)]. Histopathological analysis of OPN-/-Fas^lpr/lpr^ spleens showed a subversion of the parenchymal architecture by a monomorphic proliferation of large elements, with abundant cytoplasm and central nuclei, and immunoblastic morphology, suggestive of diffuse large B cell lymphoma with morphology resembling ABC-DLBCL (Fig. 1B). Flow cytometry analysis of splenic B cells with the classical B cell markers, CD19, B220 and IgM, showed in 5/6-month-old OPN-/-Fas^lpr/lpr^ mice a population of CD19 + cells expressing variable levels of B220 and low to negative expression of surface IgM *(*Fig. 1C). These cells were rarely detectable in OPN-competent Fas^lpr/lpr^ spleens*.*

### Lymphomas developing in OPN-/-Fas^lpr/lpr^ mice show features of ABC-DLBCL

**Fig. 2:**
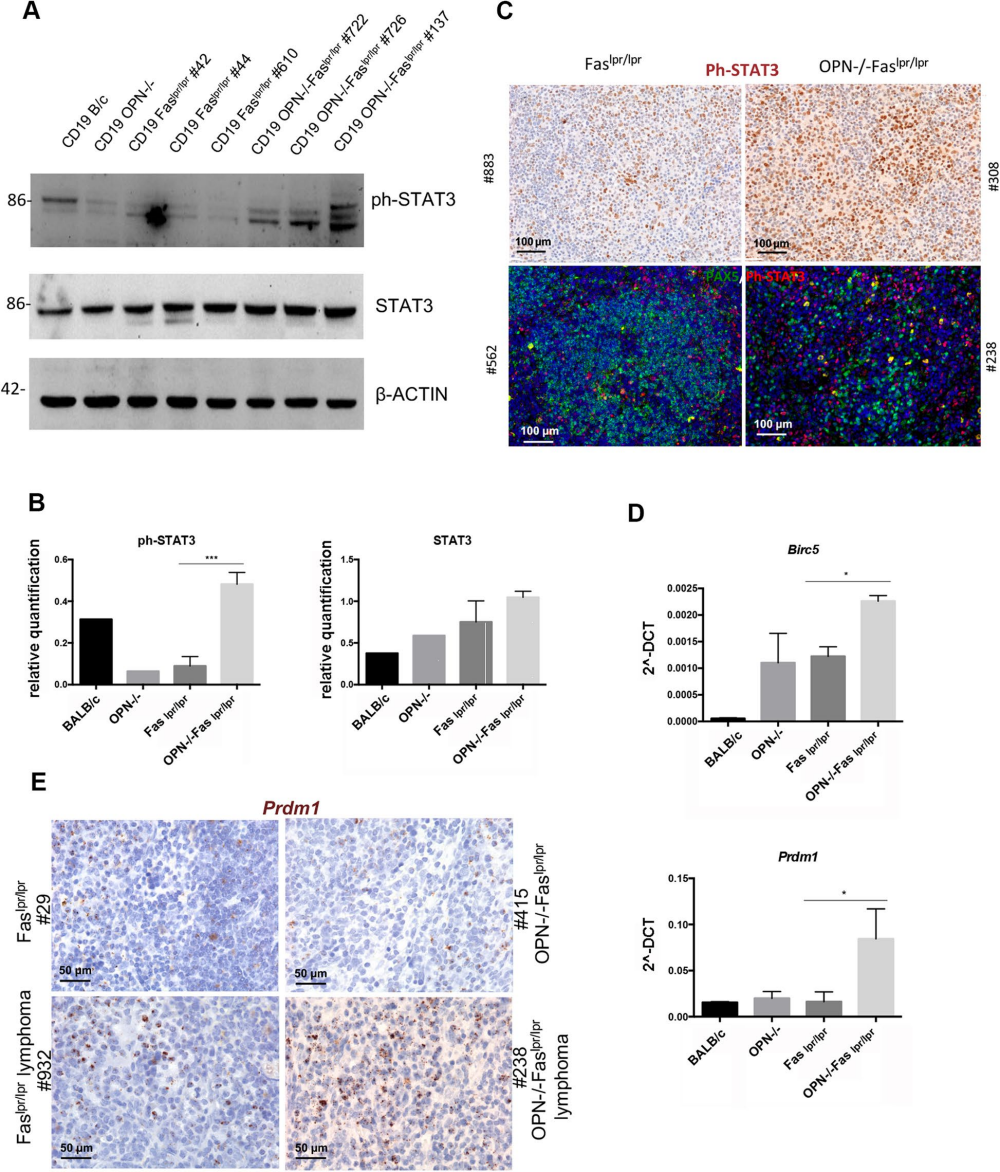
Evaluation of STAT3 pathway activation in CD19 + B cells from autoimmune mice. **A**. Western blot illustrating the expression of phospho- and total STAT3 in splenic CD19 + B cells from Fas^lpr/lpr^ (*n = *3) and OPN-/-Fas^lpr/lpr^ (*n = *3) mice. CD19 + from a BALB/c and a OPN-/- mouse were used as controls. **B**. Quantification of western blot, relative to β-ACTIN as housekeeping gene of ph-STAT3 (Student t test; ***, p:0.0007) and total STAT3 (ns, p: 0.1237). Statistics was applied to values related to Fas^lpr/lpr^ and OPN-/-Fas^lpr/lpr^ B cells. **C**. Representative IHC (upper panels) showing the expression of ph-STAT3 in OPN-sufficient and -deficient lymphomas. Scale bar: 100 μm. Representative IF (lower panels) for PAX5 (pan B cell marker) and ph-STAT3 on OPN + / + and -/-tumours. Scale bar: 50 μm. **D**. RT-PCR on purified splenic CD19 + cells from Fas^lpr/lpr^ and OPN-/-Fas^lpr/lpr^ mice showing the expression of STAT3- target genes *Prdm1 (*Student t test; *, p: 0.0261***)*** and *Birc5 (*Student t test; *, *p*: 0.0195*)*. CD19 + from B/c and OPN-/- mice were used as controls. Values are shown as 2^-DCT. Data are a pool of two independent experiments with 3 samples for each group. **E**. Representative RNA scope imaging showing *Prdm1* expression on spleens from autoimmune mice

Being the different subtypes of DLBCL, GCB- and ABC-type, classified according to the cell of origin and by specific and distinguishable protein expression [7], we tested their key molecular features in OPN-sufficient and -deficient B cell lymphomas. Tumors developed in Fas^lpr/lpr^ mice were positive for BCL6, a marker generally expressed by human GC-related DLBCLs, whereas lymphomas developed in absence of OPN were negative for this marker (Fig. 1D, upper panels). These lymphomas were also characterized by a high expression of the proliferation marker Ki67 (Fig. 1D, lower panels), quantitatively confirmed by flow cytometry analysis (Fig. 1E). The lymphoma type-specific protein landscape of splenic CD19 + B cells of SLE-prone mice was analyzed by western blot for the expression of BCL2, that together with c-MYC defines the class of “double expressor” lymphomas, and for IRF4, an additional marker belonging to the ABC-DLBCL signature. BCL2 and IRF4 were significantly more expressed in OPN-deficient than -competent Fas^lpr/lpr^ CD19 + cells, further supporting the histopathological assessment of high-grade ABC-DLBCL lymphomas (Fig. 1F-G). We also observed in CD19 + cells derived from OPN-deficient spleens a striking activation of STAT3 (Ph-STAT3), a key pathway for B cell survival and proliferation (Fig. 2A-B). This finding was confirmed in situ by Ph-STAT3 immunohistochemical and immunofluorescence staining (Fig. 2C) and by the higher expression of the STAT3 target genes, *Prmd1* and *Birc5,* in CD19 + cells from OPN-deficient Fas^lpr/lpr^ mice (Fig. 2D). A strong *Prmd1* expression in OPN-deficient lymphomas was further validated by RNA Scope technology (Fig. 2E). Altogether, these data indicate that lymphomas developing in autoimmunity-prone mice, in absence of OPN, are ABC-DLBCLs, potentially of the “double-expressor” type.

To gain some insight into the B cell subset undergoing malignant transformation in OPN-/-Fas^lpr/lpr^ mice, spleens from OPN-competent and -deficient naïve and Fas^lpr/lpr^ mice were analyzed by flow cytometry using Hardy’s gating strategy (Fig. S2A) [30] to distinguish the different B cell subpopulations. Interestingly, we noticed that, inside the mature CD19 + CD93- population, which comprises the classical follicular (FOB) (CD23 +) and marginal zone (MZB) (CD21/35 +) B cell subsets, OPN-/-Fas^lpr/lpr^ mice showed a significant reduction of FOB fraction in favor of an “atypical” CD23-CD21/35- population (Fig. S2B). In line with the speculation that candidates this atypical population for undergoing transformation in OPN-/-Fas^lpr/lpr^ mice, we confirmed in situ*,* by IHC, that OPN-deficient lymphomas were negative for these markers (Fig. S2C).

### Gene expression profile on CD19 + B cells confirmed the ABC-DLBCL phenotype of OPN-/-Faslpr/lpr tumours

**Fig. 3:**
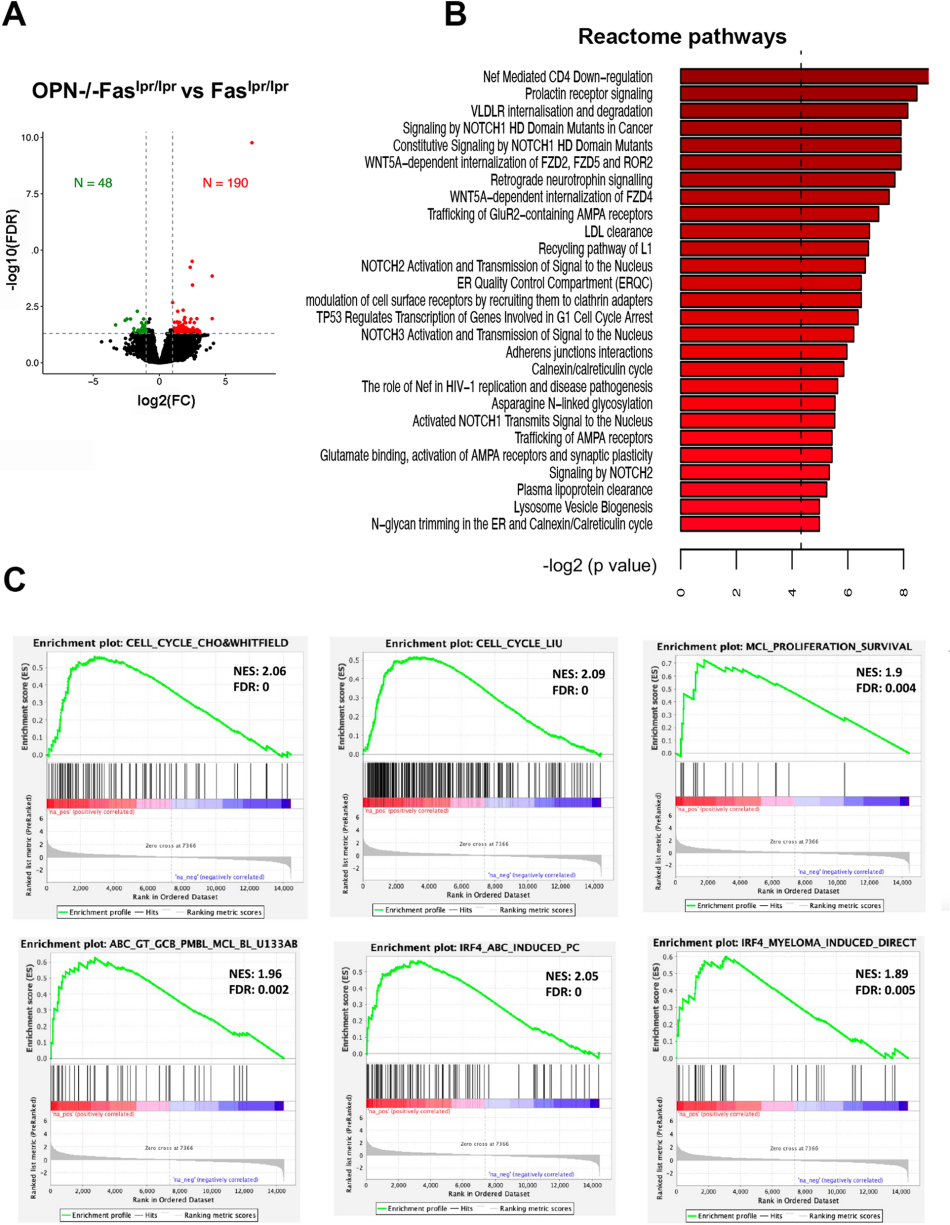
Gene expression profile of CD19 + cells from OPN-deficient and –sufficient animals. **A**. Volcano plot showing the up- and down-regulated genes comparing splenic CD19 + cells from OPN-/-Fas^lpr/lpr^ and Fas^lpr/lpr^ mice. **B**. PathfindR analysis on Reactome pathway illustrating the modulated signaling pathways in the comparison between OPN-/-Fas^lpr/lpr^ and Fas^lpr/lpr^ B cells. **C**. GSEA functional annotation of OPN-/-Fas^lpr/lpr^ vs Fas^lpr/lpr^ B cells gene expression data

Aiming at investigating which genes and pathways could drive lymphomagenesis in OPN-/- Fas^lpr/lpr^ mice, we performed gene expression profiling (GEP) on CD19 + B cells, purified through magnetic beads from the spleen of Fas^lpr/lpr^ and tumour-bearing OPN-/-Fas^lpr/lpr^ mice at 5 months of age, when OPN-deficient autoimmune mice already show evident signs of lymphomas. We identified 190 genes significantly up-regulated (log_2_FC ≥ 1 and FDR < 0.05) and 48 significantly down-regulated genes (log_2_FC ≤ 1 and FDR < 0.05) in OPN-/-Fas^lpr/lpr^ mice compared to Fas^lpr/lpr^ (Fig. 3A and Supplementary table S1). Among up-regulated genes, we interestingly found *Prdm1* [a STAT3-target gene already found enriched in OPN-/-Fas^lpr/lpr^ B lymphocytes (Fig. 2D-E)], *Prlr* and *Hsp90b1*, all genes that are highly expressed and/or amplified in human ABC-DLBCL samples [31–33]. Then, to evaluate whether B cells from Fas^lpr/lpr^ and OPN-/-Fas^lpr/lpr^ mice could activate different signaling cascades, thus reflecting their commitment to transformation, we performed pathway analysis on the list of differentially expressed genes. We found that OPN-/-Fas^lpr/lpr^ B cells up-regulated pathways relevant for B cell development and often hyper-activated in several B cell malignancies, including DLBCL, such as NOTCH pathways [34], that, of note, is induced downstream the TLR-mediated activation of STAT3 [35] (Fig. 3B). Importantly, the same DEGs and de-regulated pathways were not found in the comparison between OPN-deficient and –sufficient naïve control mice (Supplementary table S2).

To assess whether our murine data find correlation with human lymphomas, we performed GSEA applying our list of DEGs between 5-month old OPN-/-Fas^lpr/lpr^ and Fas^lpr/lpr^ mice to different lymphoma-related signatures and found an enrichment in signatures associated with cell cycle progression and cell proliferation, as well as ABC-DLBCL categorization and IRF4 activation, confirming our results obtained with the spontaneous mouse model (Fig. 3C).

### B cell intracellular OPN restrains TLR9-MYD88 signaling pathway

**Fig. 4:**
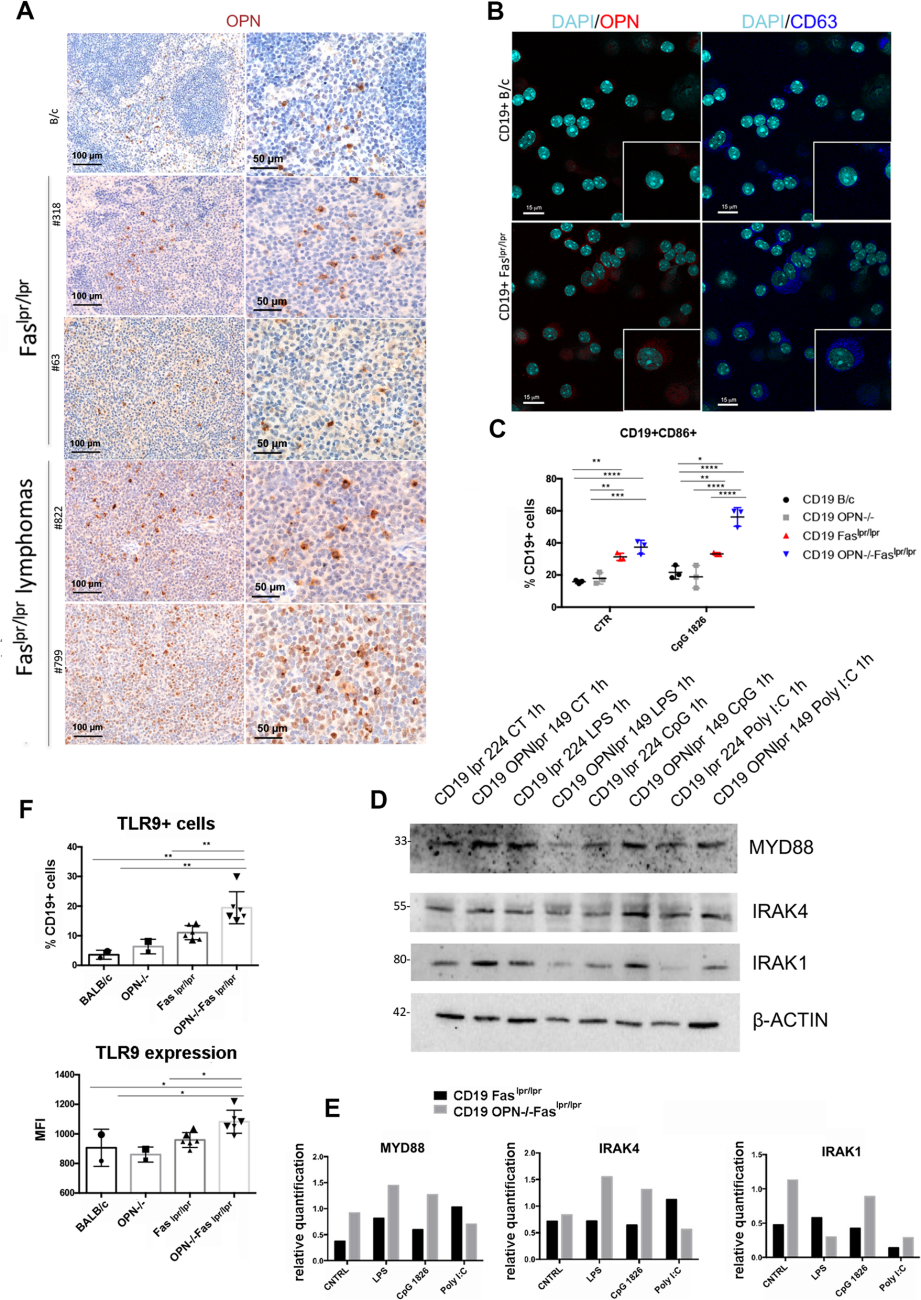
Expression of OPN and evaluation of TLR9-MYD88 signaling pathway in CD19 + cells from autoimmune mice*.* **A**. IHC for OPN performed on splenic tissues from BALB/c and autoimmune mice with and without lymphomas. Scale bar: 100 μm (left) and 50 μm (right). **B**. Representative double IF for OPN (red) and the endosomal marker CD63 (blu) on purified CD19 + cells from BALB/c and Fas^lpr/lpr^ mice. Scale bar: 15 μm. **C.** Flow cytometry analysis showing CD86 expression on CD19 + cells from naïve and autoimmune mice (*n = *3) with or without 3-day stimulation with CpG 1826 (*, *p <* 0.05, **, *p <* 0.01, ***, *p <* 0.001, ****, *p <* 0.0001, Two-way ANOVA). **D**. Representative western blot showing the level of MYD88, IRAK4 and IRAK1 in CD19 + B cells purified from Fas^lpr/lpr^ and OPN-/-Fas^lpr/lpr^ mice at steady state condition and after TLR9-triggering with CpG 1826. The TLR4 agonist LPS and the TLR3 ligand Poly I:C were used as positive and negative controls, respectively. **E**. Western blot quantification relative to β-ACTIN housekeeping gene of MYD88, IRAK4 and IRAK1. **F**. Flow cytometry analysis showing the percentage of CD19 + TLR9 + cells (upper panel) and TLR9 protein level (MFI) on CD19 + cells (lower panel) from the spleen of Fas^lpr/lpr^ (*n = *6) and OPN-/-Fas.^lpr/lpr^ mice (*n = *6). B/c and OPN-/-mice were used as controls. Data in the graphs are referred to a pool of two independent experiments. (**, *p <* 0.01; Ordinary one way ANOVA) (*, *p <* 0.05; Ordinary one way ANOVA)

To understand whether B-cell intrinsic OPN could contrast lymphomagenesis in Fas^lpr/lpr^ mice, we first investigated its expression in splenic B cell zones of autoimmune mice. Notably, IHC for OPN showed a moderate expression in B cells of the spleen follicles in Fas^lpr/lpr^ mice without tumours, that greatly increased in lymphomatous B cells from tumour-bearers (Fig. 4A and S3). Of note, OPN expression was localized at the edge of the nuclear region, suggesting that the OPN expressed by lymphomatous cells is mainly intracellular. Accordingly, double IF performed on CD19 + cells purified from Fas^lpr/lpr^ mice showed a similar expression pattern of OPN and CD63, an endosomal marker (Fig. 4B), suggesting that, in autoimmune B cells, OPN is mainly intracellular and localized in the endosomal compartment.

In light of this result, to identify intrinsic molecular mechanisms that could drive B cell proliferation and transformation in autoimmune mice, we investigated the activation of TLR9-MYD88 signaling pathway, since TLR9 can bind autoimmune dsDNA and MYD88 has been described to co-localize with, and to be regulated by, iOPN although in cells different from B lymphocytes [17–19]. Additionally, *MYD88* activating mutations are predominant drivers in human ABC-DLBCL [36]. We therefore isolated by immunomagnetic beads CD19 + cells from the spleen of 4-month-old OPN-deficient and -sufficient mice, to be stimulated with the TLR9 agonist CpG 1826. While naive B cells did not show any significant difference upon stimulation, flow cytometry analysis indicated that CD19 + B cells from OPN-/-Fas^lpr/lpr^ mice were more activated, based on the increased expression of CD86, compared to OPN-sufficient autoimmune B cells, upon TLR9 triggering (Fig. 4C).

In agreement, the protein level of molecules belonging to MYD88 signaling, such as IRAK1, IRAK4, and MYD88 itself, were clearly up-regulated in B cells deficient for OPN, both in steady state conditions and after stimulation with CpG 1826 (Fig. 4D-E), suggesting that the presence of OPN can restrain MYD88 signaling pathway. Consistent with the MYD88-iOPN crosstalk, also stimulation with TLR4 agonist LPS up-regulated MYD88 itself and IRAK4 in OPN-deficient autoimmune B cells (Fig. 4D-E), whereas TLR3 (MYD88-independent) agonist Poly I:C, used in this experimental setting as negative control, did not induce similar increased activation (Fig. 4D-E). These results confirm that the negative role exerted by B cell-intrinsic OPN likely affects only MYD88-dependent TLR cascades.

The enhanced activation of MYD88 could also be due to an increased expression of TLR9 in OPN-deficient cells, an event confirmed by flow cytometry (Fig. 4F). Indeed, TLR9 expression was striking up-regulated in CD19 + B cells from 5/6-month-old OPN-/-Fas^lpr/lpr^ mice in comparison with age-matched OPN competent animals, both in terms of percentage of CD19 + TLR9 + cells and mean fluorescence intensity (MFI) (Fig. 4F).

Together, these findings indicate that the TLR9-MYD88 signaling is enhanced in B cells from OPN-/-Fas^lpr/lpr^ mice. Such increase can be due both to the releasing of the brake “ iOPN”, which localized in the endosomal compartment together with TLR9, and to the higher expression of TLR9 in OPN-deficient B cells from autoimmune animals.

### Intracellular OPN prevents CpG-mediated STAT3 and/or NF-κB activation in autoimmunity-driven lymphoma cellular models

**Fig. 5:**
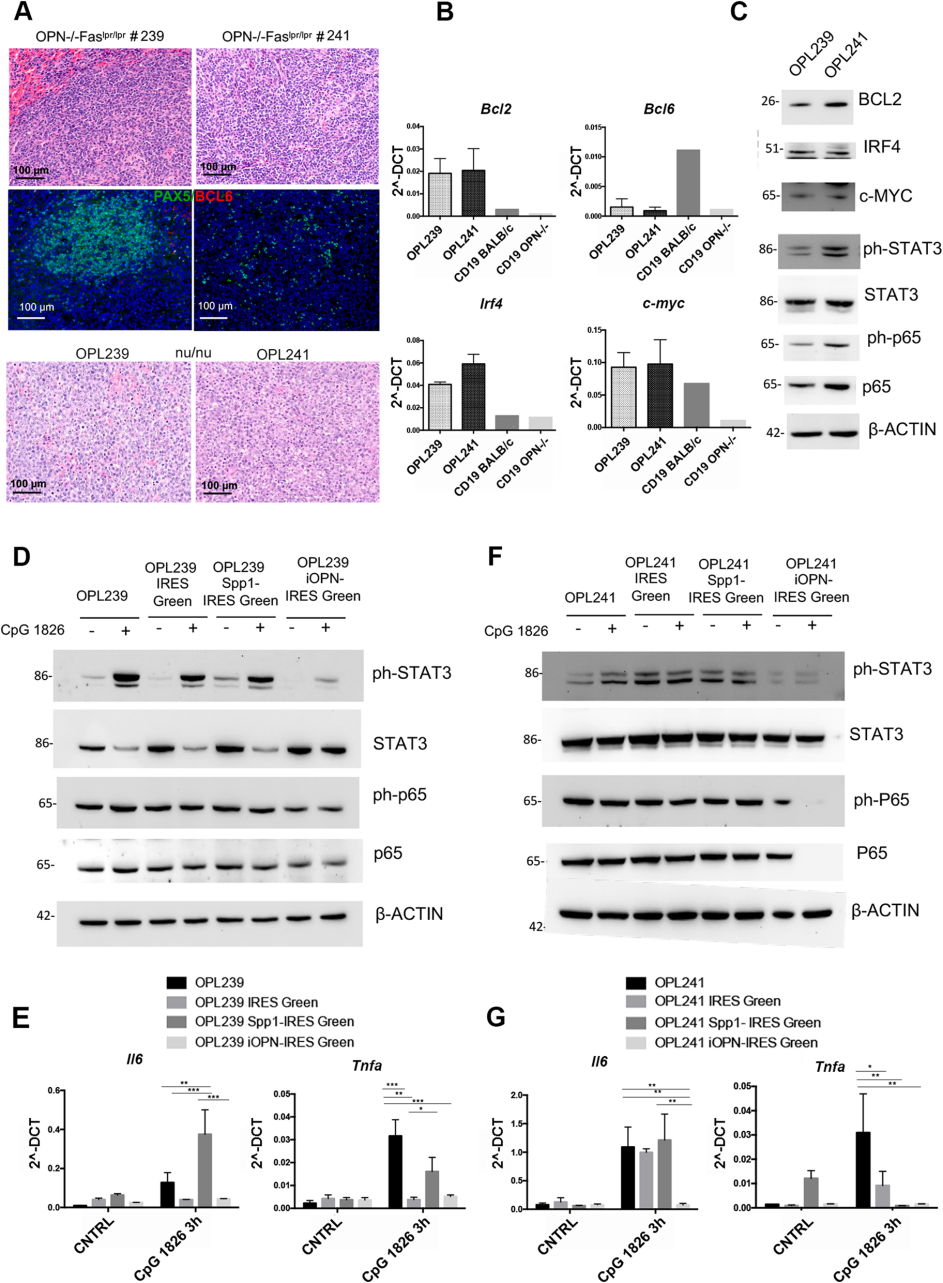
Over-expression of sOPN and iOPN in OPN-/-Fas^lpr/lpr^ DLBCL cell lines and evaluation of TLR9-MYD88 cascade. **A**. H&E (upper panels) and IF for PAX5/BCL6 (middle panels) performed on OPN-/-Fas^lpr/lpr^ mouse spleens from which OPL239 and OPL241 derive. H&E staining on tumorigenic spleens from nu/nu mice intravenously injected with OPL239 and OPL241 (lower panels). Scale bar: 100 μm. **B**. RT-PCR showing the expression of *Bcl6*, *Bcl2*, *Irf4* and *c-myc* in OPL239 and OPL241 cell lines. CD19 + from BALB/c and OPN-/-mice were used as controls. Values are shown as 2^-DCT. **C**. Western blot on OPL239 and OPL241 lysates illustrating the level of BCL2, c-MYC, IRF4 and the basal level/activation of STAT3 and NF-κB (p65) pathways. **D**. Western blot for phospho- and total STAT3, phospho- and total p65 (NF-κB) performed on OPL239, OPL239 IRES-Green, OPL239 Spp1-IRES-Green and OPL239 iOPN-IRES-Green with or without 30-min stimulation with CpG 1826. **E**. RT-PCR for *Il6* and *Tnfa* on OPL239 cell variants with or without 3 h-stimulation with CpG 1826. Values are shown as 2^-DCT. (*, *p <* 0.05; Two-way ANOVA), (**, *p <* 0.01; Two-way ANOVA), (***, *p <* 0.001; Two-way ANOVA). **F**. Western blot on OPL241 variants illustrating the expression of phospho- and total STAT3 and NF-κB with or without 30-min stimulation with CpG 1826. **G.** RT-PCR for *Il6* and *Tnfa* on OPL241 cell variants with or without 3 h-stimulation with CpG 1826. Values are shown as 2^-DCT. (*, *p <* 0.05; Two-way ANOVA), (**, *p <* 0.01, Two-way ANOVA)

To study in vitro the molecular mechanisms behind the phenotype observed in the spontaneous mouse models, we established two cell lines, named OPL239 and OPL241, from a lymphomatous spleen of two 6 month-old OPN-/-Fas^lpr/lpr^ mice. According to histopathological evaluation and immunofluorescence staining for PAX5 of the original spontaneous lymphomas, OPL239 was derived from an early-stage DLBCL that still preserved the nodular architecture, whereas OPL241 originated from a more advanced DLBCL, already spread in the spleen (Fig. 5A, upper and middle panels). When injected into nu/nu mice, both cell lines were tumorigenic, producing morphologically similar lymphomas (Fig. 5A, lower panels). Additionally, both cell lines maintained in vitro the phenotype observed in the spontaneous lymphomas in vivo, with complete loss of surface IgM and positivity for B220 (Fig. S4A). Since B cell lymphomas are usually B cell receptor (BCR)-dependent, although lymphomatous B cells can occasionally survive without BCR signaling [37], we evaluated by flow cytometry the expression of other immunoglobulins, and observed that both cell lines maintained the expression of IgA, but not of IgD (Fig. S4A), suggesting that they may still preserve intact the BCR signaling. Furthermore, RT-PCR showed that both cell lines express low levels of *Bcl6* and high levels of *Irf4/Mum1, Bcl2* and *c-myc* (Fig. 5B). Protein expression of IRF4, BCL2 and c-MYC was confirmed by Western blot (Fig. 5C). We also evaluated in the two cell lines the activation of STAT3 and NF-κB pathways by western blot and found that OPL241 line shows the strongest expression of phospho- and total p65, ph-STAT3, and BCL2 (Fig. 5C), in agreement with the high histological grade of the original lymphomas. In line with our hypothesis that the transforming B cell subset was an atypical CD21/35^−^CD23^−^ population, we assessed whether OPL239 and OPL241 maintained the same characteristics, and indeed they were negative for CD21/35 and expressed very low level of CD23 (Fig. S4B).

These results indicate that OPL239 and OPL241 cell lines well recapitulate the phenotype of ABC-DLBCL spontaneously developing in OPN-deficient autoimmune mice, representing suitable models to study in depth the role of OPN in autoimmunity-driven lymphomas.

Therefore, to assess which isoform of OPN is relevant in protecting Fas^lpr/lpr^ mice from lymphomagenesis, we over-expressed in OPL239 and OPL241 cell lines either the full-length, which encodes for the secreted OPN (sOPN), or its intracellular isoform (iOPN), using lentiviral vectors carrying, in addition to the specific OPN cDNA, a GFP reporter sequence (Spp1-IRES-Green and iOPN-IRES-Green, respectively). We confirmed the transcript level of *Spp1* through RT-PCR (Fig. S4D) and the protein level of OPN, with or without the presence of Brefeldin A (BFA) to block protein secretion, through western blot (Fig. S4E).

To mimic the pathophysiological scenario present in autoimmune mice in which dsDNA constantly circulates in the blood and triggers TLR9, we firstly confirmed TLR9 expression in both lines (Fig. S3C), and then stimulated them with CpG 1826. Remarkably, we found that the activation of STAT3 (ph-STAT3) was almost completely abolished in iOPN-overexpressing OPL239, indicating that the presence of iOPN inhibited the TLR9-MYD88-STAT3 signaling, whereas secreted OPN did not alter the physiological activation of this pathways observed in control cells (Fig. 5D and Fig.S4F, upper panels). Differently, the NF-κB pathway was not significantly modulated by 30-min CpG stimulation in all cell variants, although the overall level of both total and ph-p65 protein was slightly decreased in iOPN-overexpressing cells (Fig. 5D and Fig.S4F, upper panels). To confirm the inhibitory effect of iOPN, we assessed the expression of TLR9-MYD88-downstream genes, after a 3 h-stimulation and we found that OPL239 up-regulated, or not, *Il6* and *Tnfa* upon TRL9-triggering if transduced to over-express soluble or intracellular iOPN gene, respectively (Fig. 5E), in line with immunoblot results.

On the other hand, OPL241 cell line and its variants seemed less sensitive to TLR9 stimulation than OPL239, as none of them showed significant activation of both STAT3 and p65 pathways, with only a slight induction of ph-STAT3 in the parental OPL241 line (Fig. 5F and Fig.S4F, lower panels). Interestingly, we found a complete absence of both total and phospho-p65 in OPL241 iOPN-IRES-Green after TLR9-stimulation (Fig. 5F and Fig.S4F, lower panels), indicating that iOPN likely blocks this pathway also at the transcriptional level. Furthermore, the phosphorylation of STAT3 was reduced in iOPN-overexpressing cells, regardless the stimulus (Fig. 5F and Fig.S4F, lower panels). Consistent with immunoblot results, *Il6* expression was up-regulated at 3 h time point in OPL241 over-expressing *Spp1* at the same level of control cells, while iOPN-overexpressing cells did not show any increased expression (Fig. 5G). Only the parental OPL241 line up-regulated *Tnfα* expression upon TLR9 triggering, whereas its levels were not modified in any of the genetically-modified variants (Fig. 5G). A possible explanation for the different results obtained with OPL241 variants could be a baseline higher MYD88-madiated STAT3 and NF-κB activation in these cells, as already described in Fig. 5C.

These results overall mechanistically demonstrate that iOPN represent a brake in TLR9-MYD88 signaling pathways in autoimmunity-driven B cell lymphomas, likely explaining why in OPN-deficient Fas^lpr/lpr^ mice the transformation of B cells occurs more frequently than in OPN-competent counterparts.

### Over-expression of secreted and intracellular OPN exerts different effects in vivo

Wondering whether the over-expression of either sOPN or iOPN in lymphoma cells could affect their behaviour in vivo, we intravenously injected OPL239-IRES-Green, Spp1-IRES-Green and iOPN-IRES-Green cells in sub-lethally irradiated BALB/c mice. We chose OPL239 as the model for in vivo experiments since this line derives from an early-stage lymphoma (Fig. 5A), thus likely more susceptible to sOPN or iOPN restoration.

**Fig. 6:**
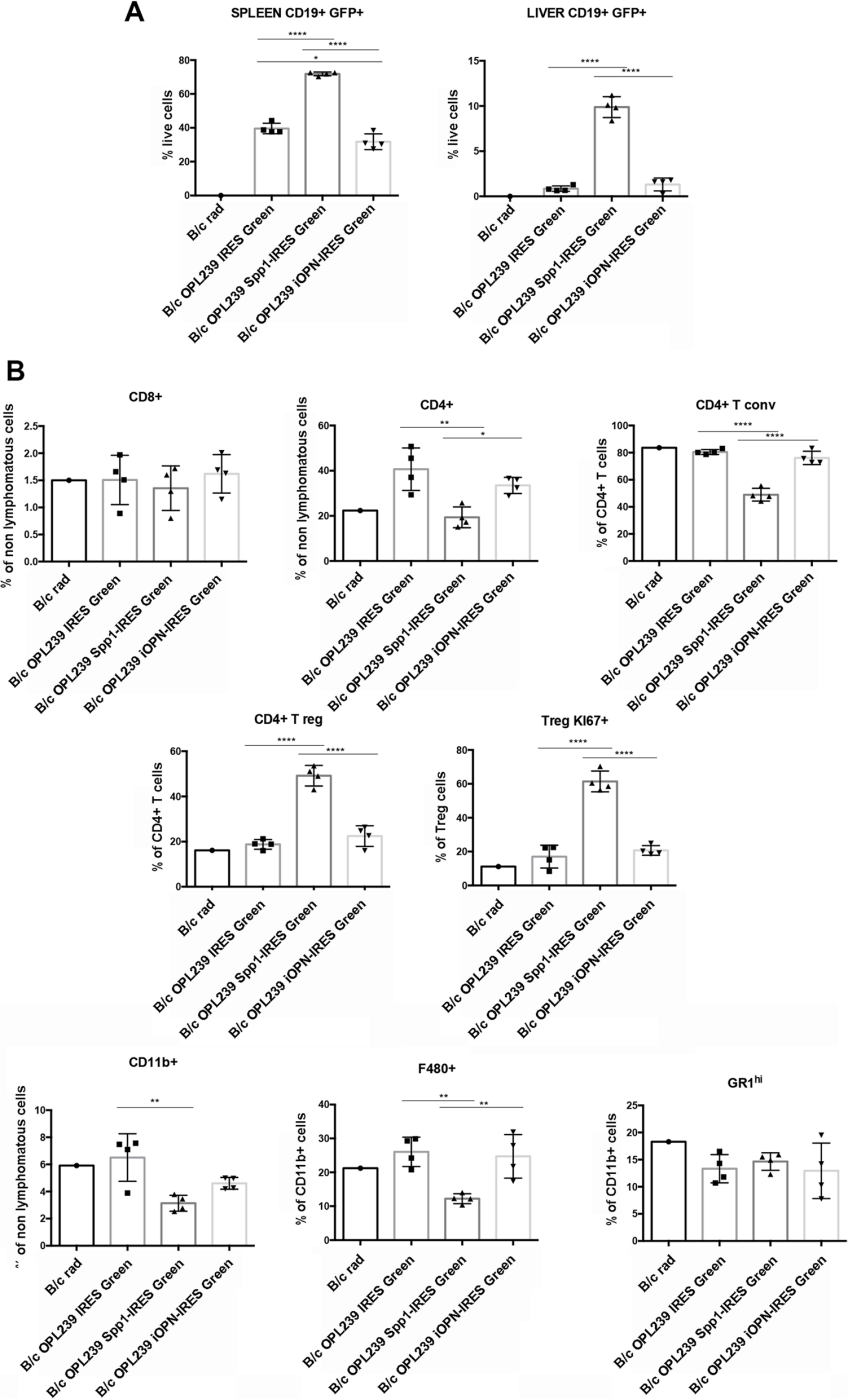
In vivo effects of over-expression of the soluble and the intracellular OPN in OPN-/-Fas^lpr/lpr^ DLBCL cells. **A**. Flow cytometry analysis showing the tumour take (% of CD19 + GFP +) in spleen and liver of 5 Gy irradiated BALB/c mice injected with OPL239 IRES-Green, OPL239 Spp1-IRES-Green and OPL239 iOPN-IRES-Green. Four mice per group were used in the experiment (*, *p <* 0.05; ****, *p <* 0.0001; Ordinary one way ANOVA). **B**. Flow cytometry analysis showing the percentage of lymphoid and myeloid populations in the spleen of mice injected with OPL239 cell variants [(T conv: T conventional cells; T reg: T regulatory cells) (*, *p <* 0.05, **, *p <* 0.001; ****, *p <* 0.0001; Ordinary one way ANOVA)]. An irradiated B/c mouse was used as control

As shown in Fig. 6A, in line with a pro-tumoral role of secreted OPN, OPL239 Spp1-IRES-Green cells showed a significant increase in tumor take, assessed as CD19 + GFP + population in the spleen, in comparison to control and iOPN-overexpressing cells. Interestingly, iOPN-over-expressing cells showed slight but significant reduction in tumor take in the spleen compared with OPL239-IRES-Green control cells (Fig. 6A). These results indicate that iOPN not only does not exert the pro-tumorigenic activities as secreted OPN does, but also that it could be able to restrain DLBCL growth. In line with the increased tumor take in case of cells over-expressing the secreted OPN, we found an overall reduction in total CD4 T cells that were, however, significantly enriched in Foxp3 + T regulatory cells, that were also highly proliferative, in comparison to control and iOPN-over-expressing cells (Fig. 6B). No significant difference was detected for CD8 T cells, whereas total myeloid cells were reduced in case of secreted OPN-over-expressing cells, likely due to a reduction in F4/80 + macrophages (Fig. 6B).

Altogether, these results confirm that the over-expression of secreted OPN by lymphoma cells induces an aggressive phenotype, associated to a tolerant microenvironment, allowing tumour cells to proliferate and colonize organs and indicate that, on the contrary, iOPN does not exert such effects but rather, it may restrain inflammation and possibly tumor growth.

### OPN is down-regulated in human ABC-DLBCL in comparison with GC-DLBCL patient samples and shows an intracellular localization

**Fig. 7:**
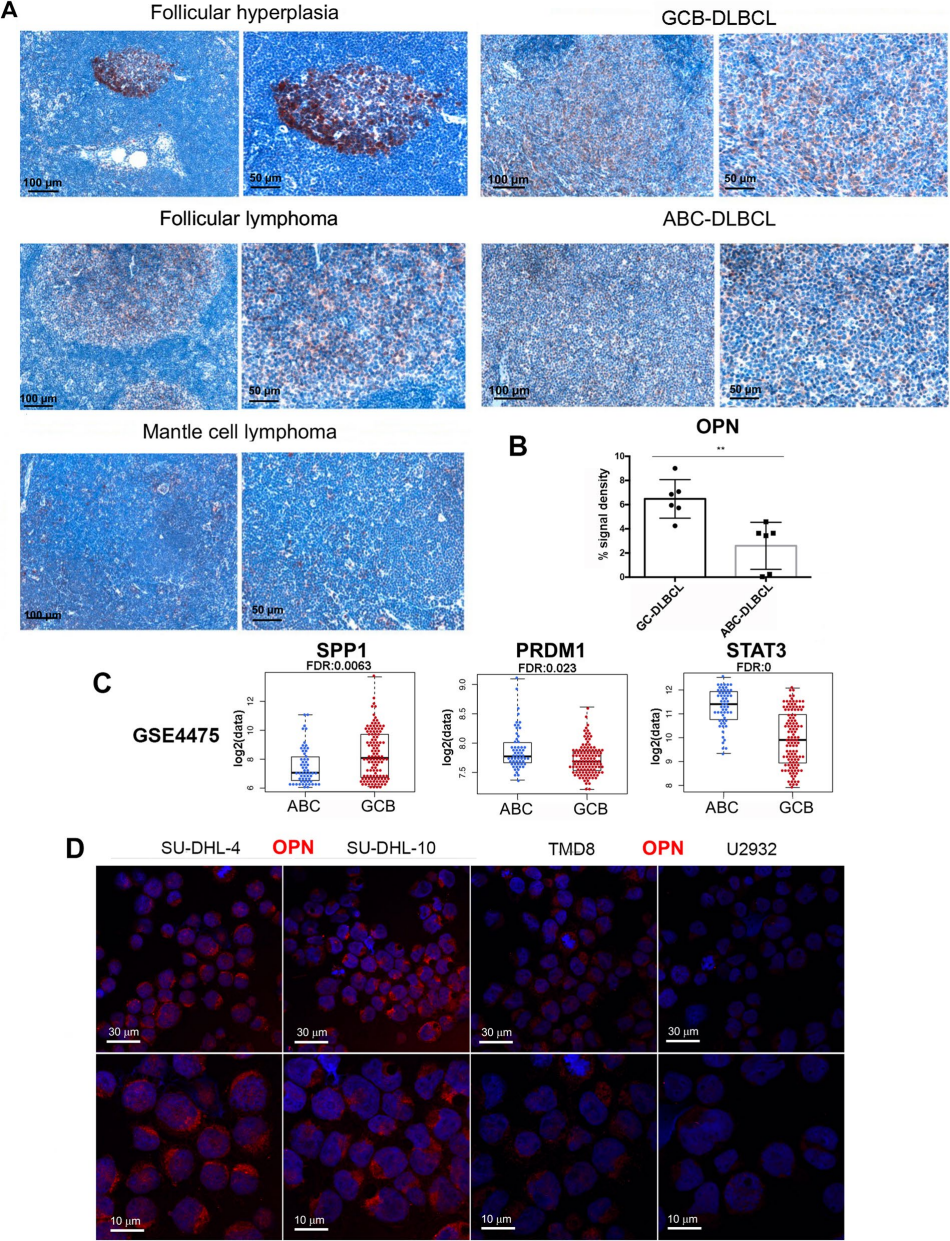
Evaluation of OPN expression in human DLBCL samples and cell lines. **A**. Representative IHC for OPN performed on human lymphoma biopsies. Scale bar: 100 μm (left) and 50 μm (right). **B**. Quantification of OPN expression on GC- vs ABC-DLBCL samples (Student t test; **, p: 0.0036). Six cases were analyzed for each subtype. Quantification was performed analysing 5 areas/sample by using Positive Pixel count v9 Leica Software Image Analysis algorithm. **C**. Boxplots showing the expression of *SPP1* (Student t test; *** *p:* 0.0009)*, PRDM1* (Student t test, **, *p*: 0.0047) and *STAT3* (Student t test, ****, *p*: 4.863E-14) in ABC- VS GCB-DLBCL samples in GSE4475. Dots in boxplot represent the expression of the selected gene in each sample. **D**. Immunofluorescence on cytospin preparation showing OPN expression in SU-DHL-4 and SU-DHL-10 (GC-DLBCL-s), TMD8 and U2932 (ABC-DLBCL) cell lines. Scale bar: 30 μm (upper panels) and 10 μm (lower panel)

To assess the translation relevance of our findings and the potential role of OPN in human DLBCLs, we first analyzed its expression by IHC on biopsies derived from patients affected by different haematological diseases: follicular hyperplasia, follicular and mantle cell lymphomas, GC- and ABC-DLBCL. The highest expression of OPN was detected in biopsies of follicular- and germinal center-related diseases, whereas it was poorly expressed in ABC-DLBCL (Fig. 7A). To strengthen this result we analyzed six cases of GCB and ABC DLBCL subtype, confirming a significantly lower OPN expression in the latter and suggesting that low/absence of OPN may facilitate the occurrence of ABC-DLBCL, in line with the OPN-deficiency described in our spontaneous mouse model (Fig. 7B and Fig.S5).

Also, taking advantage of a publicly available dataset (GSE4475 [38]) comprising 221 DLBCL clinical specimens (58 ABC-, 120 GCB-DLBCL, 43 samples unclassifiable), we observed that *SPP1* was downregulated in ABC- versus GCB-DLBCL (FC: 1.75, FDR: 0.0063) (Fig. 7B), confirming the IHC data. Additionally, in line with the mouse data, *PRDM1* and *STAT3* expression was significantly higher (FC: 1.12, FDR: 0.023 and FC: 2.53, FDR: 0, respectively) in ABC- than GCB-DLBCL clinical specimens (Fig. 7B).

These results indicate that OPN-/-Fas^lpr/lpr^ lymphomas recapitulate to some extent the biology of human ABC-DLBCL showing similar phenotype and gene expression of specific markers, opening the issue of whether, also in human setting, OPN may exert a protective role. To support this hypothesis, we explored whether also human DLBCL cell lines do express the intracellular form of OPN by performing IF for OPN on cytospin preparation of SU-DHL-4, SUDH-L-10 (GC-DLBCL human cell lines), TMD8 and U2932 (ABC-DLBCL human cell lines). As shown in Fig. 7C, OPN is evidently less expressed in the two ABC-type cell lines, in agreement with our data on both murine model and human tissues. It is noteworthy that the localization of the protein is mostly intracellular, accumulated at the peri-nuclear/endosomal region of the cells (Fig. 7C), a pattern very similar to that found in Fas^lpr/lpr^ B lymphocytes (Fig. 4B).

## Discussion

Systemic Lupus Erythematosus (SLE) and other autoimmune disorders are associated with an increased risk of malignancies, particularly lymphoma [3–5]. However, the lymphoma pathogenesis of SLE patients remains not completely understood. We look at the autoimmune-prone Fas^lpr/lpr^ mouse model that develops a SLE-like syndrome and, upon aging, B cell lymphomas, to study the process of lymphomagenesis in an autoimmune contexture.

In this study we demonstrate that in Fas^lpr/lpr^ mice the intracellular form of osteopontin (iOPN) is protective against the development of B cell lymphomas. Indeed, Fas^lpr/lpr^ animals rendered OPN-deficient (OPN-/-Fas^lpr/lpr^ mice) showed a twofold increase of splenic DLBCL in comparison with OPN-sufficient counterparts. In addition, all lymphomas developed in OPN-/-Fas^lpr/lpr^ mice were of the ABC-DLBCL type based on the expression of BCL2, C-MYC, IRF4 and of high proliferation rate (Ki67), in absence of BCL6. Differently, the fewer lymphomas developed in Fas^lpr/lpr^ animals showed a strong positivity for BCL6, a classical marker of human GCB-DLBCLs, and negligible expression of the ABC-related proteins. Notably, we observed a striking STAT3 activation in OPN-deficient lymphomas, suggesting that OPN-/-Fas^lpr/lpr^ tumors could rely on this pathway to survive.

OPN-deficient autoimmune mice showed in the splenic CD93^−^ mature population, which includes the follicular (FOB, CD23^+^) and the marginal zone (MZB, CD21/35^+^) B cell subsets, a significant increase of the CD23^−^CD21/35^−^ fraction. This fraction, generally neglected in the classification of splenic B cell subsets, is potentially the population that transforms under the pressure of autoimmune condition. Our result suggests that OPN may act as a brake to the expansion of this “atypical” B cell population. In agreement, the two lymphoma cell lines (OPL239 and OPL241) we have derived from lymphomatous spleens of OPN-/-Fas^lpr/lpr^ mice were characterized to have the CD23^−^CD21/35^−^ phenotype, along with all other markers of ABC-DBLCL.

In mice, OPN is present in two isoforms: the secreted and the intracellular (iOPN) forms, the latter never being described in B cells. Here, we show the intracellular form in autoimmune splenic B cells, accumulated as dots surrounding the peri-nuclear region. Of note, its expression is increased in the lymphomatous areas. Immunofluorescence analysis showed that iOPN and the endosomal marker CD63 display the same pattern of expression, further supporting the idea that iOPN exerts its function in the endosomal compartment, where TLR9 also localizes and signals.

Our data indicate that B cell-intrinsic iOPN can prevent the exacerbation of autoimmunity-driven B cell proliferation and activation, through the impairment of MYD88 signaling cascade. Indeed, we found a significant up-regulation of TLR9 in splenic CD19 + B cells from OPN-/-Fas^lpr/lpr^ mice that could likely increase the binding of the circulating autoimmune double strand DNA and its pathway activation. Accordingly, we observed an increase of the MYD88 protein machinery (i.e. IRAK1, IRAK4 and MYD88) either in steady state conditions or after stimulation with the TLR9 agonist CpG 1826 in OPN-deficient autoimmune B cells compared to OPN-sufficient counterpart.

These findings are in line with works demonstrating anti-inflammatory properties of iOPN in the TLR cascades of myeloid cells [18, 19]. The co-localization between iOPN and the scaffold protein MYD88 has been documented in a few reports [17, 18], although with different outcomes. One report shows that iOPN promotes MYD88-mediated IFNα production in plasmacytoid dendritic cells [17], while the other one demonstrates how replacing iOPN in OPN-/- macrophages suppressed NF-κB target cytokines [18]. Disregarding the iOPN-MYD88 co-localization, another paper shows that iOPN negatively controls TLR4-mediated inflammation by regulating GSK3β and 4EBP1 phosphorilation [19]. Our results are the first showing the presence and the anti-inflammatory role of iOPN in autoimmune B lymphocytes.

Further evidence of the anti-inflammatory role exerted by iOPN were obtained by overexpressing either iOPN or the secreted forms (sOPN) in the two DLBCL cell lines we have developed. Indeed, after TLR9 triggering with CpG 1826, only the iOPN-transduced cells were able to inhibit pathways relevant for B cell survival and activation, i.e. STAT3 and NF-κB. Accordingly, intravenous injection of OPL239 cells transduced with sOPN showed increased tumor growth in compariosn with control mock-transduced cells, whereas injection of iOPN-expressing cells showed slightly reduced tumor take, in agreement with our idea of iOPN restraining B cell proliferation and activation.

Our data indicate that B cell-intrinsic iOPN contrasts/prevents lymphomagenesis in Fas^lpr/lpr^ mice exerting a negative feed-back loop on STAT3 and NF-κB signaling cascade, a process otherwise promoted in its absence (OPN-/-mice). Nevertheless, 33% of Fas^lpr/lpr^ mice still develop B cell lymphomas in an OPN-competent setting, suggesting that, in mice characterized by a high inflammation associated with autoimmune condition, the endogenous iOPN is not sufficient to completely halt B cell transformation. The increased expression of iOPN detected in B cells from lymphomatous areas of Fas^lpr/lpr^ mice, in comparison to naïve mice, may represent an unsuccessful attempt to limit STAT3 and NF-κB signaling activation.

The association between low/absent OPN and DLBCL of the ABC type was confirmed in human patients by immunostaining comparing DLBCL biopsies and cell lines of ABC- versus GC-types. Importantly, we also demonstrated, by immunofluorescence analysis on human DLBCL lines of the GC-type, an intracellular localization of OPN accumulated in the peri-nuclear endosomal compartment, mirroring the findings on mouse autoimmune B cells. Of note, a nuclear localization of OPN has been demonstrated in the humans Rck8 DLBCL lymphoma cell line from central nervous system, sustaining NF-κB activation through the down-regulation of its inhibitors [21]. These different findings suggest that a specific intracellular compartmentalization of OPN (endosomal versus nuclear) could lead to distinct outcome.

Complementing our previous findings on the development of a CD5 + B cell malignancy in absence of SPARC [9], we show here that, under the same lymphoproliferative spur of Fas^lpr/lpr^ autoimmune-prone setting, the deficiency of a different protein of the same family, OPN, favors the onset of a different type of B cell malignancy. Interestingly, while in the first setting the molecular mechanisms responsible for B cell transformation were microenvironment-related due to the disarrangement of the SLO stromal network that promoted the redistribution and pathogenic interaction of CD5^+^ B cells and neutrophils, in this work the mechanism underlying the transition to B cell lymphomagenesis is mainly B cell-intrinsic, with intracellular OPN restraining TLR9-MYD88 signaling and therefore acting as a brake to B cell proliferation and activation. Our studies underlie the importance of exploring the complex dynamics characterizing lymphomagenesis through functional experiments, probing new players of cell intrinsic and/or microenvironment-related mechanisms. Here, we are adding a new piece to the mosaic of the spontaneous development of an ABC-like DLBCL showing the role of a non-canonical “oncosuppressor”, such as iOPN, and enforcing the concept that lymphomagenesis can occur beyond the activity of strong oncogenic drivers, prompting new investigation on other molecules that may favor/accelerate lymphoma development.

Although we have clearly demonstrated that the defective expression of intracellular OPN in the lymphoid tissue undergoing remodeling, due to persistent immune stimulation, is a determinant of malignant B cell transformation and acquisition of an ABC-like DLBCL phenotype, our study does not allow envisaging direct or short-term influences on DLBCL diagnosis, prognostication, or treatment. In this context, whereas the classical “secreted” OPN has always been associated to a tumor-promoting activity, and, in line, in our study it sustains the aggressiveness of an already established lymphoma cell line, by identifying intracellular OPN as a relevant “brake” in the process of DLBCL lymphomagenesis, this work raises a “warning” in considering OPN, straightforwardly, a bad prognostic factor or a potential therapeutic target. Indeed, considering such different role/activity of the intracellular and secreted forms of OPN we have here unveiled in the context of DLBCL lymphomas, but that may possibly be in place also in other tumor settings, a very careful design for clinical prognistication or intervention using OPN should be considered.

## Conclusions

In summary, we demonstrate here that the intracellular isoform of OPN, localizing in the endosomal compartment of autoimmune-prone B lymphocytes, dampens DNA-triggered, MYD88-mediated activation of STAT3 and NF-κB pathways, thus limiting DLBCL development. According to mouse findings, we also discovered a highly expressed endosomal OPN in human cell lines of the GC-DLBCL subtype, which, in comparison with the ABC-DLBCL counterpart, is a less aggressive tumor, suggesting that, also in the human setting, the intracellular isoform of OPN may limit the aggressiveness of the disease.

## Supplementary Information

**Additional file 1.** **Supplementary file 1.** Supplementary Material & methods.

**Additional file 2:** **Supplementary Table S1.** Differentially expressed genes between CD19+ cellsfrom OPN-/-Fas lpr/lpr and Faslpr/lpr mice.

**Additional file 3:** **Supplementary Table S2.** Differentially expressed genes between CD19+ cellsfrom OPN-/-and OPN+/+ mice.

**Additional file 4:** **Supplementary Figure S1.** Evaluation of autoimmunity in Fas^lpr/lpr^ and OPN-/-Fas^lpr/lpr^ mice. **A**. Quantification of OPN in sera from Fas^lpr/lpr^ mice at 2 (*n=*8) and 5 months of age (*n=*7) by ELISA. Sera from BALB/c and OPN-/- mice were tested as controls. Data are expressed as ng/ml and are a pool of 2 experiments (*, *P<*0.05; Ordinary one way ANOVA). **B**. Flow cytometry analysis showing the relative number of splenic autoimmune CD3+B220+ T cells in Fas^lpr/lpr^ (*n=*15) and OPN-/-Fas^lpr/lpr^ mice (*n=*18) and at about 5-6 months of age. The graph shows a pool of 3 different experiments (***, *P<*0.001; Student t test). **C**. Representative spleen photograph from BALB/c, OPN-/-, Fas^lpr/lpr^ and OPN-/-Fas^lpr/lpr^ mice. D. Representative H/E staining of spleen samples from 5 month-old Fas^lpr/lpr^ and OPN-/-Fas^lpr/lpr^ mice. Reactive lymphoid cells in OPN-competent animals and initial lymphomatous foci in OPN-deficient counterparts are shown by black arrows, respectively. Magnification 20x (left) and 40x (right). **E**. Flow cytometry analysis showing the relative number of peripheral blood autoimmune CD3+B220+ T cells in and Fas^lpr/lpr^ (*n=*6) and OPN-/-Fas^lpr/lpr^ mice (*n=*7) at about 5-6 months of age. The graph refers to one representative experiment (*, *P<*0.05; Student t test).

**Additional file 5:** **Supplementary Figure S2.** Evaluation of the different spenic B cell subsets. **A**. Example of Hardy’s gating strategy to discern the different CD93+ immature (Transitional T1, T2, T3) and CD93- mature [follicular B (FOB), marginal zone B (MZB) and CD21/35-CD23-] B cell subsets in the spleen from a BALB/c mouse. **B**. Flow cytometry analysis based on Hardy’s multiparametric panel illustrating the fraction of splenic CD23+ FOB, CD21/35+ MZB cells, and CD23-CD21/35- cells from the spleens of naive and autoimmune mice. 3 mice per group were used for the experiment. Data are referred to one representative experiment out of 3 (***, *P<*0.001; Two-way ANOVA) (****, *P<*0.0001; Two-way ANOVA). **C**. Representative IHC performed on OPN-sufficient and –deficient tumours for CD23 FOB and CD21 MZB markers.

**Additional file 6:** **Supplementary Figure S3.** Immunohistochemistry staining of OPN IHC for OPN was performed in Fas ^lpr/lpr^ and OPN-/-Fas ^lpr/lpr^ mice with either no lymphoma or with lymphomatous cells. As expected, no staining is detected in case of OPN-deficient mice.

**Additional file 7:** **Supplementary Figure S4.** Characterization of OPL239 and OPL241 DLBCL cell lines. **A**. Flow cytometry analysis showing the expression of B220, IgM, IgD and IgA in OPL239 and OPL241 cell lines. **B**. Hardy’s multiparametric flow cytometry panel illustrating the expression of CD93, CD21/35 and CD23 on OPL239 and OPL241 cell lines. **C**. Flow cytometry analysis showing the expression of TLR9 on OPL239 and OPL241 cell lines. **D**. RT-PCR analysis showing *Spp1* mRNA level in overexpressing cell variants. **E**. Western blot for OPN protein expression (in presence or not of BFA, that blocks protein secretion) in parental and IRES-Green-based cell variants. 4T1 mammary cell line was used as positive control. **F**. Quantification of western blot analysis shown in figure 5D and F.

**Additional file 8:** **Supplementary Figure S5**. Expression of OPN in human GCB- and ABC-DLBCL samples. Immunohistochemistry analysis for OPN was performed on six cases for GCB- and ABC-DLBCLs. Representative images for two cases for each subtype are shown (quantification is shown in Figure 7B). Magnification 20X.

## Data Availability

The datasets generated during the current study are available in the GEO repository, with accession number GSE193790 (https://www.ncbi.nlm.nih.gov/geo/query/acc.cgi?acc=GSE193790).

## Abbreviations

ABC: Activated B cell
BFA: Brefeldin A
BSA: Bovine serum albumin
DEG: Differentially-expressed genes
DEL: “Double expressor” lymphoma
DLBCL: Diffuse large B cell lymphoma
dsDNA: Double strand DNA
FBS: Fetal bovine serum
FC: Fold change
FDR: False discovery rate
FFPE: Formalin-Fixed Paraffin-Embedded
FOB: Follicular B cells
GC: Germinal center
GFP: Green fluorescent protein
GSEA: Gene set enrichment analysis
Gy: Gray
H&E: Hematoxylin and eosin
IF: Immunofluorescence
IHC: Immunohistochemistry
IFN: Interferon
iOPN: Intracellular OPN
LPR: Lymphoproliferation
LPS: Lipopolysaccharide
MFI: Mean fluorescent intensity
MZB: Marginal zone B cells
NHL: Non-Hodgkin lymphoma
OPN: Osteopontin
RT-PCR: Real time-PCR
sOPN: Secreted OPN
SLE: Systemic Lupus Erythematosus
SLO: Secondary lymphoid organ
T conv: Conventional T cells
T reg: T regulatory cells
TLR: Toll-like receptor
WB: Western blot
