# Supplementary material for: Intracellular osteopontin protects from autoimmunity-driven lymphoma development inhibiting TLR9-MYD88-STAT3 signaling
Source: Mol Cancer. 2022 Dec 12;21:215. doi: 10.1186/s12943-022-01687-6 (PMC9743519; doi:10.1186/s12943-022-01687-6)
Supplement: Supplementary file 1 — Additional file 1. Supplementary file 1. Supplementary Material & methods. [file 12943_2022_1687_MOESM1_ESM.docx]

**Supplementary Material & methods**

*Table 1. List of antibodies for flow cytometry characterization of B cells and DLBCL cell lines*

| **Marker** | **Fluorochrome** | **Company** | **Clone** | **Catalogue n°** |
| --- | --- | --- | --- | --- |
| CD19 | FITC | BD | CL1D3 | 557398 |
| B220 | PE | BD | RA3-6B2 | 561868 |
| IgM | PE-Cy7 | eBioscience | Eb121-15f9 | 25-5890-82 |
| CD3 | APC | eBioscience | 145-2c11 | 17-0031-82 |
| TLR9 | FITC | eBioscience | M9.D6 | 11-909382 |
| CD86 | PE | eBioscience | GL1 | 12-0862-82 |
| Ki67 | PE-Cy7 | eBioscience | solA15 | 25-5698-82 |
| IgD | BV510 | BD | 11-26c.2A | 563110 |
| IgA | FITC | BD | c10-3 | 559354 |

*Table 2. List of antibodies for flow cytometry characterization of B cell subsets and DLBCL cell lines*

| **Marker** | **Fluorochrome** | **Company** | **Clone** | **Catalogue n°** |
| --- | --- | --- | --- | --- |
| IgD | FITC | eBioscience | 11-26c | 11-5993-82 |
| CD23 | PE | eBioscience | B3B4 | 12-0232-82 |
| CD21/35 | PerCP-Cy5.5 | BioLegend | 7E9 | 123416 |
| CD93 | APC | eBioscience | AA4.1 | 17-5892-82 |
| CD19 | APC-eFluor780 | eBioscience | Eb101D3 | 47-0193-82 |
| CD3 | BV510 | BD | 145-2c11 | 563024 |

*Table 3. List of antibodies for flow cytometry characterization of splenic immune cell subsets*

| **Marker** | **Fluorochrome** | **Company** | **Clone** | **Catalogue n°** |
| --- | --- | --- | --- | --- |
|  |  |  | N418 |  |
| CD11b | PE | TONBO | M170 | 50-0112-U100 |
| CD3 | PerCP-Cy5.5 | TONBO | 145-2c11 | 65-0031-U100 |
| F4/80 | PE-Cy7 | BioLegend | BM8 | 123114 |
| B220 | APC | eBioscience | RA3-6B2 | 17-0452-82 |
| Gr-1 | BV510 | BD | RB6-8C5 | 563040 |
| CD8 | PE | TONBO | 2.43 | 50-1886-U025 |
| FOXP3 | PerCP-Cy5.5 | eBioscience | FJK-165 | 45-5773-82 |
| CD3 | BV510 | BD | 145-2c11 | 563024 |
| CD4 | BV650 | BD | RM4-4 | 740-446 |
| CD3 | FITC | eBioscience | 145-2c11 | 11-0031-82 |
| CD8 | PE-Cy7 | TONBO | 2.43 | 60-1886-U100 |
| Ki67 | APC | BioLegend | 16A8 | 652405 |

*Table 4. List of antibodies for immunohistochemistry and immunofluorescence*

| **Antibody** | **Company** | **Clone** | **Catalogue n°** |
| --- | --- | --- | --- |
| Rat α-mouse BCL6 | Abcam | 7D1 | ab243150 |
| Rabbit α-mouse CD21 | Abcam | SP186 | ab240987 |
| Rabbit α-mouse CD23 | Invitrogen | SP23 | MA5-14572 |
| Rabbit α-mouse Ki67 | Abcam | Polyclonal | ab15580 |
| Rabbit α-mouse OPN | Abcam | EPR21138 | ab218237 |
| Rabbit α-mouse PAX5 | Abcam | EPR3730 | ab109443 |
| Rabbit α-ph-STAT3 (Tyr 705) | Millipore | EP2147Y | 04-1059 |
| Rabbit α-human OPN | Abcam | Epr3688 | ab91655 |

CST*: Cell Signaling Technologies

*Table 5. List of RT-PCR probes*

| **Gene name** | **Company** | **ID probe** |
| --- | --- | --- |
| *Gapdh* | IDT | Mm.PT.39.a1 |
| *Bcl2* | IDT | Mm.PT.58.7362966 |
| *Bcl6* | IDT | Mm.PT.58.32669842 |
| *Birc5* | AB | Mm01261895_m1 |
| *c-myc* | IDT | Mm.PT.58.13590978 |
| *Irf4* | IDT | Mm.PT.58.31041885 |
| *Spp1* | IDT | Mm.PT.58.43709208 |
| *Gapdh* | AB | Mm99999915_91 |
| *Il6* | AB | Mm00446190_m1 |
| *Prdm1* | AB | Mm00476128_m1 |
| *Tnfα* | AB | Mm00443258_m1 |

*Table 6. List of antibodies for western blot analysis*

| **Antibody** | **Company** | **Clone** | **Catalogue n°** |
| --- | --- | --- | --- |
| Rabbit α-β-ACTIN | Sigma | A2066 | 090M4758 |
| Rabbit α-BCL2 | CST* | D17C4 | 34985 |
| Rabbit α-c-MYC | CST | D84C12 | 56055 |
| Rabbit α-IRAK1 | CST | D51G7 | 45045 |
| Rabbit α-IRAK4 | CST | Polyclonal | 43635 |
| Rabbit α-IRF4/MUM1 | Abcam | Polyclonal | ab104803 |
| Rabbit α-MYD88 | Abcam | Polyclonal | Ab2064 |
| Goat α-OPN | Sigma | Polyclonal | 07635-1MG |
| Rabbit α-ph-p65 (Ser536) | CST | 93H1 | 3033 |
| Rabbit α-p65 | CST | D14E12 | #82426 |
| Rabbit α-ph-STAT3 (Y705) | CST | D3A7 | #91456 |
| Rabbit α-STAT3 | CST | 79D7 | 4904T |

*CST: Cell Signaling Technologies
