# Supplementary material for: Intracellular osteopontin protects from autoimmunity-driven lymphoma development inhibiting TLR9-MYD88-STAT3 signaling
Source: Mol Cancer. 2022 Dec 12;21:215. doi: 10.1186/s12943-022-01687-6 (PMC9743519; doi:10.1186/s12943-022-01687-6)
Supplement: Supplementary file 4 — Additional file 4: Supplementary Figure S1. Evaluation of autoimmunity in Faslpr/lpr and OPN-/-Faslpr/lpr mice. A. Quantification of OPN in sera from Faslpr/lpr mice at 2 (n=8) and 5 months of age (n=7) by ELISA. Sera from BALB/c and OPN-/- mice were tested as controls. Data are expressed as ng/ml and are a pool of 2 experiments (*, P<0.05; Ordinary one way ANOVA). B. Flow cytometry analysis showing the relative number of splenic autoimmune CD3+B220+ T cells in Faslpr/lpr (n=15) and OPN-/-Faslpr/lpr mice (n=18) and at about 5-6 months of age. The graph shows a pool of 3 different experiments (***, P<0.001; Student t test). C. Representative spleen photograph from BALB/c, OPN-/-, Faslpr/lpr and OPN-/-Faslpr/lpr mice. D. Representative H/E staining of spleen samples from 5 month-old Faslpr/lpr and OPN-/-Faslpr/lpr mice. Reactive lymphoid cells in OPN-competent animals and initial lymphomatous foci in OPN-deficient counterparts are shown by black arrows, respectively. Magnification 20x (left) and 40x (right). E. Flow cytometry analysis showing the relative number of peripheral blood autoimmune CD3+B220+ T cells in and Faslpr/lpr (n=6) and OPN-/-Faslpr/lpr mice (n=7) at about 5-6 months of age. The graph refers to one representative experiment (*, P<0.05; Student t test). [file 12943_2022_1687_MOESM4_ESM.docx]

***Supplemental file 4***

***
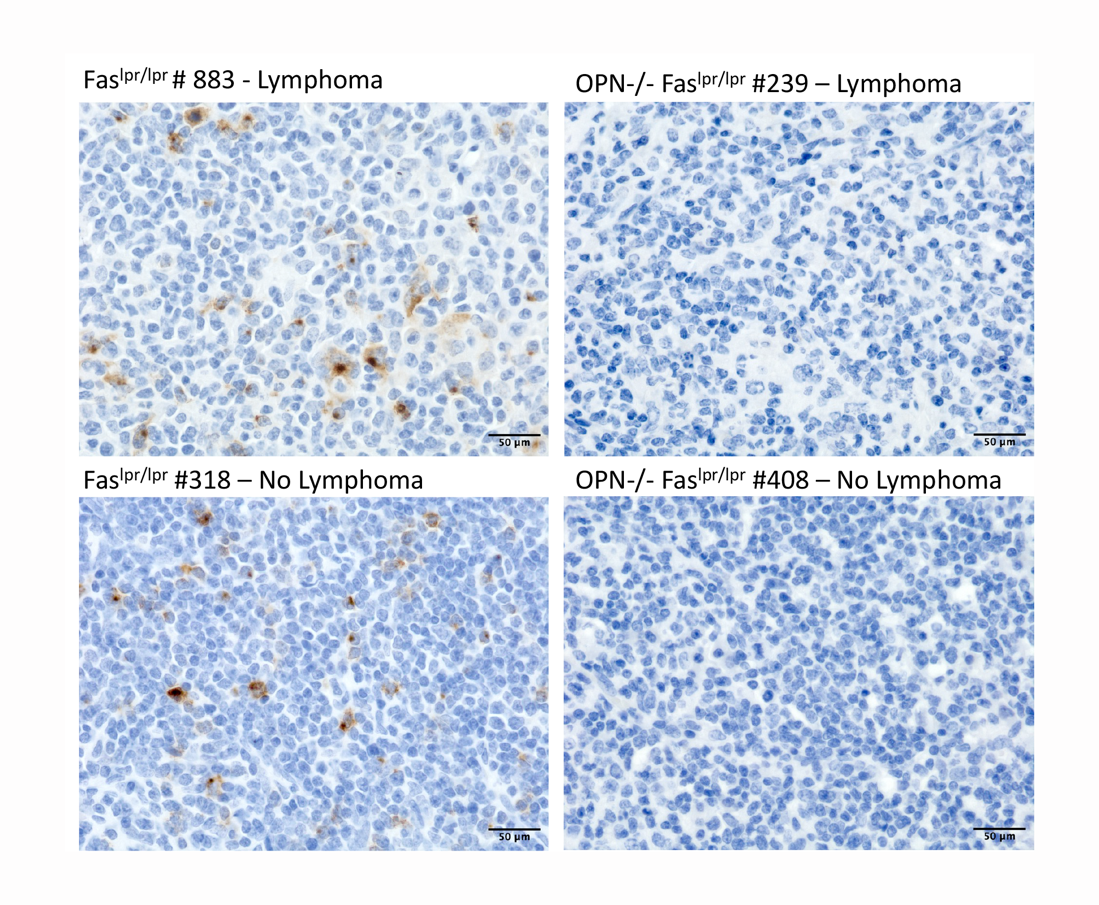
***

**Supplementary Figure S3. Immunohistochemistry staining of OPN**

IHC for OPN was performed in Fas ^lpr/lpr^ and OPN-/-Fas ^lpr/lpr^ mice with either no lymphoma or with lymphomatous cells. As expected, no staining is detected in case of OPN-deficient mice.
