# Supplementary material for: Intracellular osteopontin protects from autoimmunity-driven lymphoma development inhibiting TLR9-MYD88-STAT3 signaling
Source: Mol Cancer. 2022 Dec 12;21:215. doi: 10.1186/s12943-022-01687-6 (PMC9743519; doi:10.1186/s12943-022-01687-6)
Supplement: Supplementary file 6 — Additional file 6: Supplementary Figure S3. Immunohistochemistry staining of OPN IHC for OPN was performed in Fas lpr/lpr and OPN-/-Fas lpr/lpr mice with either no lymphoma or with lymphomatous cells. As expected, no staining is detected in case of OPN-deficient mice. [file 12943_2022_1687_MOESM6_ESM.docx]

***Supplemental file 5***

***
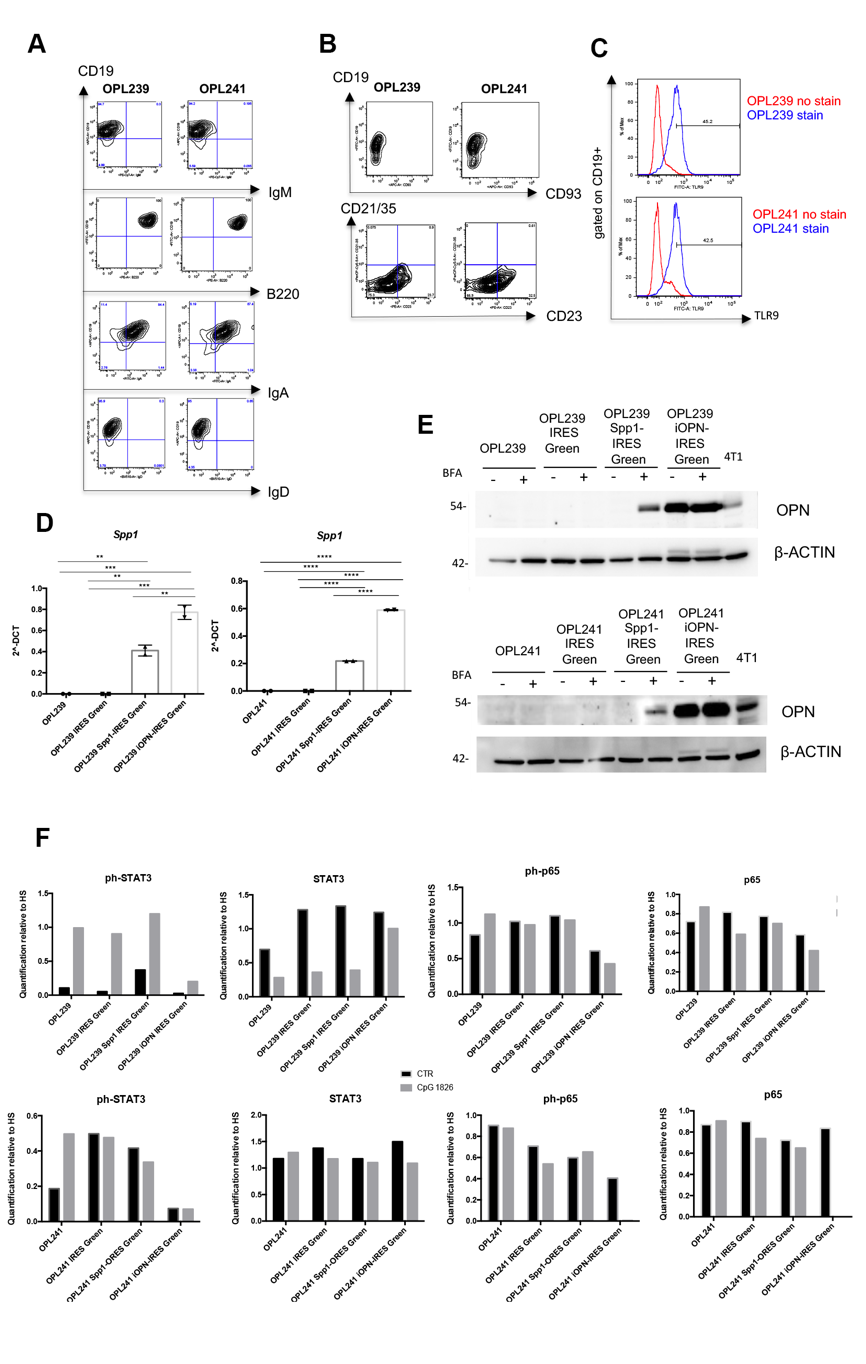
***

**Supplementary Figure S4. Characterization of OPL239 and OPL241 DLBCL cell lines. A.** Flow cytometry analysis showing the expression of B220, IgM, IgD and IgA in OPL239 and OPL241 cell lines. **B.** Hardy’s multiparametric flow cytometry panel illustrating the expression of CD93, CD21/35 and CD23 on OPL239 and OPL241 cell lines. **C.** Flow cytometry analysis showing the expression of TLR9 on OPL239 and OPL241 cell lines. **D.** RT-PCR analysis showing *Spp1* mRNA level in overexpressing cell variants. **E.** Western blot for OPN protein expression (in presence or not of BFA, that blocks protein secretion) in parental and IRES-Green-based cell variants. 4T1 mammary cell line was used as positive control. **F.** Quantification of western blot analysis shown in figure 5D and F.
