# Supplementary material for: Intracellular osteopontin protects from autoimmunity-driven lymphoma development inhibiting TLR9-MYD88-STAT3 signaling
Source: Mol Cancer. 2022 Dec 12;21:215. doi: 10.1186/s12943-022-01687-6 (PMC9743519; doi:10.1186/s12943-022-01687-6)
Supplement: Supplementary file 7 — Additional file 7: Supplementary Figure S4. Characterization of OPL239 and OPL241 DLBCL cell lines. A. Flow cytometry analysis showing the expression of B220, IgM, IgD and IgA in OPL239 and OPL241 cell lines. B. Hardy’s multiparametric flow cytometry panel illustrating the expression of CD93, CD21/35 and CD23 on OPL239 and OPL241 cell lines. C. Flow cytometry analysis showing the expression of TLR9 on OPL239 and OPL241 cell lines. D. RT-PCR analysis showing Spp1 mRNA level in overexpressing cell variants. E. Western blot for OPN protein expression (in presence or not of BFA, that blocks protein secretion) in parental and IRES-Green-based cell variants. 4T1 mammary cell line was used as positive control. F. Quantification of western blot analysis shown in figure 5D and F. [file 12943_2022_1687_MOESM7_ESM.docx]

***Supplemental file 2***


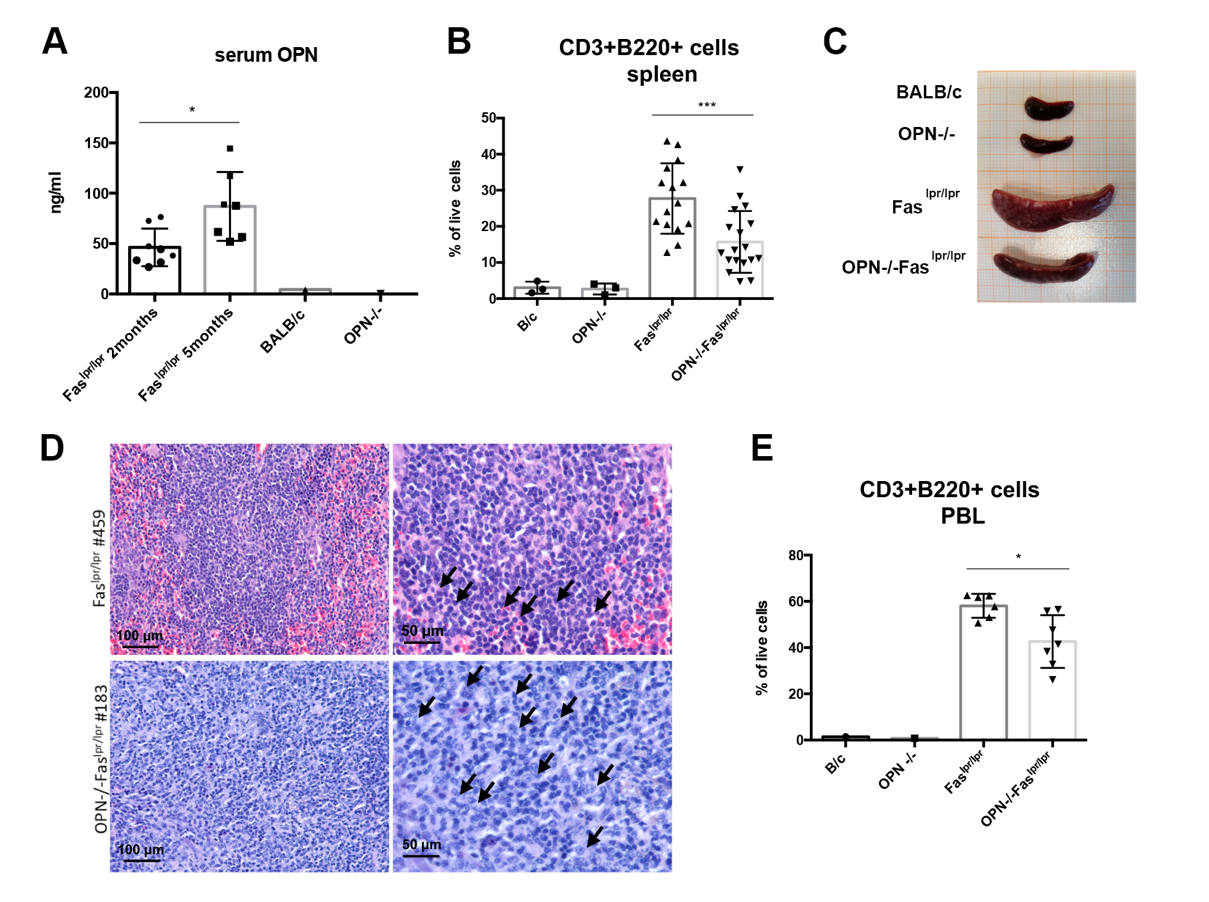


**Supplementary Figure S1. Evaluation of autoimmunity in Fas^lpr/lpr^ and OPN-/-Fas^lpr/lpr^ mice. A.** Quantification of OPN in sera from Fas^lpr/lpr^ mice at 2 (n=8) and 5 months of age (n=7) by ELISA. Sera from BALB/c and OPN-/- mice were tested as controls. Data are expressed as ng/ml and are a pool of 2 experiments (*, P<0.05; Ordinary one way ANOVA). **B**. Flow cytometry analysis showing the relative number of splenic autoimmune CD3+B220+ T cells in Fas^lpr/lpr^ (n=15) and OPN-/-Fas^lpr/lpr^ mice (n=18) and at about 5-6 months of age. The graph shows a pool of 3 different experiments (***, P<0.001; Student t test). **C.** Representative spleen photograph from BALB/c, OPN-/-, Fas^lpr/lpr^ and OPN-/-Fas^lpr/lpr^ mice. D. Representative H/E staining of spleen samples from 5 month-old Fas^lpr/lpr^ and OPN-/-Fas^lpr/lpr^ mice. Reactive lymphoid cells in OPN-competent animals and initial lymphomatous foci in OPN-deficient counterparts are shown by black arrows, respectively. Magnification 20x (left) and 40x (right). **E.** Flow cytometry analysis showing the relative number of peripheral blood autoimmune CD3+B220+ T cells in and Fas^lpr/lpr^ (n=6) and OPN-/-Fas^lpr/lpr^ mice (n=7) at about 5-6 months of age. The graph refers to one representative experiment (*, P<0.05; Student t test).
