# Supplementary material for: Intracellular osteopontin protects from autoimmunity-driven lymphoma development inhibiting TLR9-MYD88-STAT3 signaling
Source: Mol Cancer. 2022 Dec 12;21:215. doi: 10.1186/s12943-022-01687-6 (PMC9743519; doi:10.1186/s12943-022-01687-6)
Supplement: Supplementary file 8 — Additional file 8: Supplementary Figure S5. Expression of OPN in human GCB- and ABC-DLBCL samples. Immunohistochemistry analysis for OPN was performed on six cases for GCB- and ABC-DLBCLs. Representative images for two cases for each subtype are shown (quantification is shown in Figure 7B). Magnification 20X. [file 12943_2022_1687_MOESM8_ESM.docx]

***Supplemental file 3***


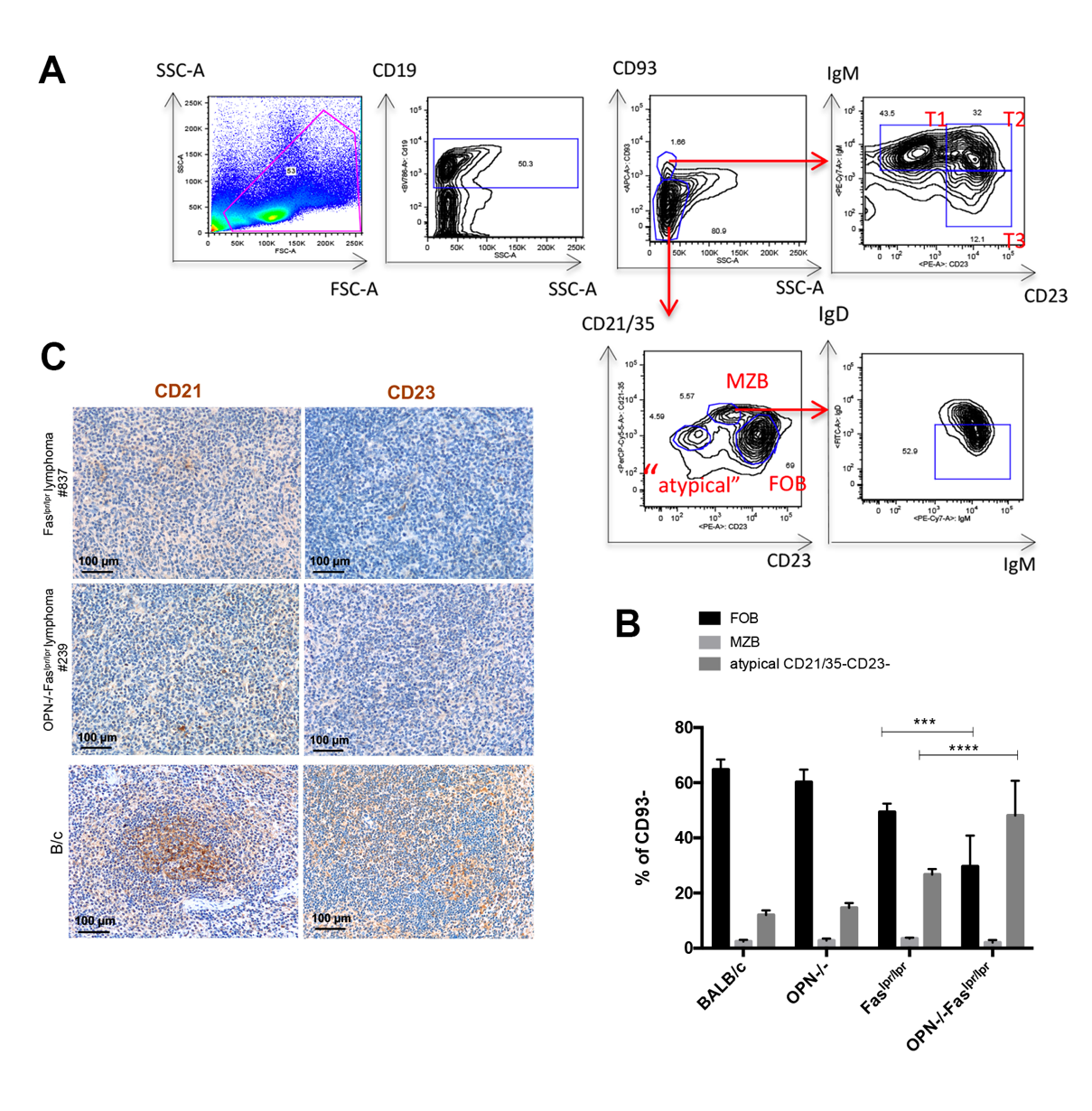


**Supplementary Figure S2. Evaluation of the different spenic B cell subsets. A.** Example of Hardy’s gating strategy to discern the different CD93+ immature (Transitional T1, T2, T3) and CD93- mature [follicular B (FOB), marginal zone B (MZB) and CD21/35-CD23-] B cell subsets in the spleen from a BALB/c mouse. **B.** Flow cytometry analysis based on Hardy’s multiparametric panel illustrating the fraction of splenic CD23+ FOB, CD21/35+ MZB cells, and CD23-CD21/35- cells from the spleens of naive and autoimmune mice. 3 mice per group were used for the experiment. Data are referred to one representative experiment out of 3 (***, P<0.001; Two-way ANOVA) (****, P<0.0001; Two-way ANOVA). **C.** Representative IHC performed on OPN-sufficient and –deficient tumours for CD23 FOB and CD21 MZB markers.
